# Hierarchical surprise signals in naturalistic violation of expectations

**DOI:** 10.1162/imag_a_00459

**Published:** 2025-01-24

**Authors:** Vincent Plikat, Pablo R. Grassi, Julius Frack, Andreas Bartels

**Affiliations:** Department of Psychology, University of Tübingen, Tübingen, Germany; Centre for Integrative Neuroscience, Tübingen, Germany; Max-Planck Institute for Biological Cybernetics, Tübingen, Germany; Rocket Magic, Tübingen, Germany

**Keywords:** surprise, magic, predictive coding, violation of expectation, intuitive physics, fMRI

## Abstract

Surprise responses signal both high-level cognitive alerts that information is missing, and increasingly specific back-propagating error signals that allow updates in processing nodes. Studying surprise is, hence, central for cognitive neuroscience to understand internal world representations and learning. Yet, only few prior studies used naturalistic stimuli targeting our high-level understanding of the world. Here, we use magic tricks in an fMRI experiment to investigate neural responses to violations of core assumptions held by humans about the world. We showed participants naturalistic videos of three types of magic tricks, involving objects appearing, changing color, or disappearing, along with control videos without any violation of expectation. Importantly, the same videos were presented with and without prior knowledge about the tricks’ explanation. Results revealed generic responses in frontal and parietal areas, together with responses specific to each of the three trick types in posterior sensory areas. A subset of these regions, the midline areas of the default mode network (DMN), showed surprise activity that depended on prior knowledge. Equally, sensory regions showed sensitivity to prior knowledge, reflected in differing decoding accuracies. These results suggest a hierarchy of surprise signals involving generic processing of violation of expectations in frontal and parietal areas with concurrent surprise signals in sensory regions that are specific to the processed features.

## 1 Introduction

Prior experience and “intuitive” knowledge about the physical world guide our perception and allow for a meaningful interaction with the environment. They set up constraints on our expectations based on what we believe to be possible in the world. For example, prior world-knowledge informs us that objects do not vanish of existence if occluded (object permanency), that objects tend to keep their features (feature constancy), that objects cannot pass through other objects (solidity), that objects do not appear out of the blue, and so forth. Informed expectations based on these intuitive physical priors allow us to quickly make sense of incoming sensory information. Such expectations have been shown to strongly modulate perception (de Lange et al., 2018). For example, a “light-from-above” prior constrains depth perception from shading (Adams et al., 2004), and knowledge that objects cannot occupy the same place at the same time explains our inability to perceive two objects simultaneously in bistable perception (Hohwy et al., 2008).

Accordingly, perception can be understood as an inferential process in which top–down informed expectations are matched with incoming sensory information (Friston, 2005,^2010^;Lee & Mumford, 2003;Rao & Ballard, 1999). In this “predictive processing” framework, deviations between expectations and incoming data (i.e., prediction errors) are used to update an internal model of the world at different levels of complexity and abstraction. Higher-level priors (like object permanency) represent more abstract aspects of the world and constrain lower-level inferences that represent more immediate features of the world (like a particular object) (Clark, 2013;Hohwy, 2014). In this context, intuitive physical knowledge can be thought of as an internal model representing different aspects of the causal structure of the physical world, not unsimilar to a “physics engine” in a virtual environment (Battaglia et al., 2013).

This prior knowledge of physical principles can be studied using events that seemingly violate them. For example, evidence from developmental psychology using so-called violation of expectation (VOE) paradigms suggest that infants acquire important aspects about the workings of the physical world during the first year of life, such as object permanency and solidity (Hespos et al., 2009;S. Wang, 2004;Wynn, 1992). However, it is largely unclear how surprise-responses relating to intuitive physical principles are represented in the human brain.

Most previous neuroimaging studies investigated responses to lower-level VOE, for example using paradigms involving novel or infrequent (and thus unexpected) stimuli (e.g.,Linden, 1999;Stevens et al., 2000;Todorovic et al., 2011;Wessel et al., 2012), omission of expected stimuli (e.g.,Todorovic et al., 2011;Wacongne et al., 2011), or changes in stimulus sequences (Downar et al., 2000,^2001^;Grundei et al., 2023). These studies have consistently revealed the involvement of lower-level stimulus-specific prediction errors in modality- and feature-specific areas (i.e., visual areas are modulated by visual surprises, auditory cortex by auditory surprises, and so forth, see e.g.,Egner et al., 2010;Kok et al., 2012;Todorovic et al., 2011), as well as of higher-level frontal and parietal areas, including the fronto-insular and anterior cingulate cortex (ACC) (as reviewed inFouragnan et al., 2018;Kim, 2014). Importantly, these frontal and parietal areas are consistently involved in signaling lower-level sensory expectation violations irrespective of sensory modality (Downar et al., 2000;Grundei et al., 2023).

In contrast, only few studies investigated VOE of higher-level physical principles (such as object permanency). These studies used more complex stimuli such as computer-generated animations (Bardi et al., 2017;Liu et al., 2024) or naturalistic videos showing magic tricks (Danek et al., 2015;Parris et al., 2009) and consistently revealed higher-level surprise signals in frontal and parietal areas. This is largely in line with the suggested role of several frontal and parietal areas in the representation of physical concepts (Fischer et al., 2016;Schwettmann et al., 2019). However, and in contrast to studies investigating lower-level VOEs, modality and feature-specific sensory areas have either not been in the focus of these higher-level VOE studies or have hitherto not observed VOE-related activity in them (cf.,Liu et al., 2024).

Here, we designed a novel VOE paradigm to uncover the effect of VOE of physical concepts (i.e., “world-model” VOEs) on both: previously identified higher-level frontal and parietal areas, as well as lower-level sensory areas. Our paradigm contained a battery of standardized magic videos that we presented to human participants while measuring fMRI responses to investigate: 1) which regions are generally involved when viewing natural videos that violate physical principles; 2) whether specific types of violations, like appearance of objects, or changes of color, modulate sensory areas known to process the feature concerned; and 3) whether knowledge of the explanation of a given magic trick modulates the observed VOE activity.

To this aim, we created and validated videos for a naturalistic VOE paradigm showing either dedicated magic tricks (to create the illusion of seemingly impossible events to actually occur, cf.,Grassi & Bartels, 2021) or matched control actions that involved no violation of physical principles. The VOE videos were designed to evoke surprise responses related to unexpected object appearance, disappearance of objects, and feature change (color-changing objects). They were performed by a professional magician (Julius Frack), and each trick-type was performed using three common objects (balls, playing cards, and pencils). Each trick was presented before and after revealing the method of the tricks. This allowed us to compare responses with and without VOE using identical videos.

Univariate analyses revealed a hierarchy of surprise signals: frontal and parietal areas, including the dorsal ACC and areas of the default mode network (DMN), were involved when perceiving events violating physical principles regardless of the type of trick used, and their activity was positively correlated with subjective surprise. In contrast, posterior sensory areas were modulated specifically by the type of expectation-violation, such as color-processing medial fusiform cortex by color change, and object-selective LOC by the appearance of objects. Controls indicate that their modulation is due to the feature-specific surprise and not due to the feature-change. Multivariate analyses extended the results: information about the specific types of expectation-violations was exclusively encoded in posterior regions, and significantly decodable down to the earliest levels of cortical visual processing (V1–V3). Additionally, decoding accuracy significantly decreased with prior knowledge, revealing a reduction in surprise signals. Together, our results demonstrate a generic response in frontal and parietal areas to violation of physical principles, along with concurrent representations of specific expected information in early sensory areas.

## 2 Methods

### 2.1 Participants

We performed fMRI on 27 subjects. Three subjects were excluded from data analysis due to excessive movement and/or sleepiness during the scanning sessions. Data from a total of 24 subjects were analyzed (16 female; 8 male; mean age 24.4 ± 4.3 SD years). All subjects had normal or corrected to normal vision, no history of neurological impairments nor contraindication for fMRI. Participants had no expertise as magicians and were naive to the magic tricks used. Participants provided written informed consent prior to the experiment. The study was approved by the ethics committee of the University Clinic Tübingen and was conducted in accord with the Declaration of Helsinki.

### 2.2 VOE stimuli

#### 2.2.1 Video recordings

A set of 63 videos were created for the VOE study. In accord with previous work (Parris et al., 2009), we created videos for three different conditions: the videos showed either magic tricks (magic condition, 18 videos), similar actions without a magic event (control condition, 18 videos), or unusual actions with the objects used in the magic tricks (unusual condition, 9 videos). We included the unusual condition to investigate neural correlates of surprise in a similar setting while not violating any physical concept (cf.,Parris et al., 2009). We further created explanation videos showing how each of the magic tricks was achieved (18 videos).

All videos were performed by professional illusionist Julius Frack in a standardized setting consisting of a black background and a black table (seeFig. 1Afor an example). To investigate the effect of specific VOE, we presented magic tricks showing three different violations of physical principles: appearances (A) (i.e., a red object appears), color changes (C) (i.e., a red object changes color to blue), and vanishes (V) (i.e., a red object disappears) (seeFig. 1C). To generalize across objects, we used three easily distinguishable objects: balls, playing cards, and thick pencils. Each object was used equally often in each trick type. Finally, we created two versions of each type of VOE for each object (e.g., two different videos showing a red ball changing to a blue ball using different methods).

**Fig. 1.**
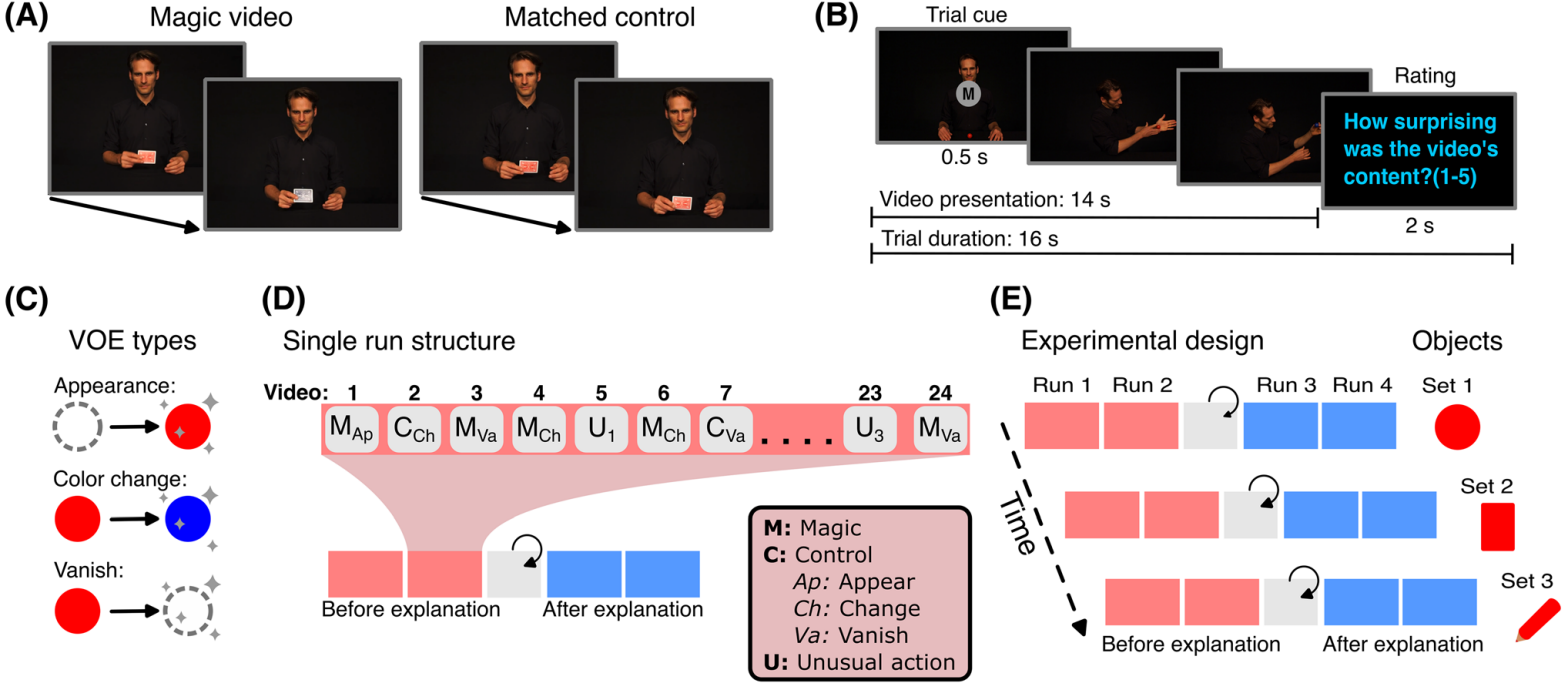
(A), example of a color-changing card magic trick (left) and its corresponding matched control video (right). (B), shown is the timeline of a single trial. Every video started with a central cue lasting 500 ms, indicating whether the video will show a magic trick (M) or not (X). Each video presentation lasted 14 s; if a video happened to be shorter than 14 s, the last frame of the video was shown until the total duration was 14 s as a static image. After the video, subjects had 2 s to answer how surprising the video’s content was. (C), shown are the three different types of VOE used (i.e., “magic events”). We created videos showing magic tricks using three objects (balls, playing cards, and pencils) that showed either an unexpected object appear (Ap), change color (from red to blue) (Ch) or vanish (Va). For each object-VOE combination, we created two tricks (i.e., we had two color-changing card tricks). (D), schematic example of a single experimental set. One set consisted of four runs—two before and two after explanation runs (i.e., pre and post revelation). Between pre- and post-revelation runs, we showed participants videos explaining the magic tricks performed during the corresponding set. Each run showed 24 videos with a pseudo-randomized order, ensuring that the same video was not presented in two consecutive trials (12 magic videos [M], 6 matched controlled videos [C], and 6 unusual actions [U]). (E), complete experimental design showing all three sets. The experiment was divided into three sets, one for each of the objects. Each set was divided into four experimental runs, where each run showed all the videos of an object but in a randomized order. After the second fMRI run, the methods behind the tricks shown in the set were revealed, dividing the runs into*before*and*after*the explanation of the tricks (pre- and post-revelation, respectively).

#### 2.2.2 Full set of stimuli

Each of these magic tricks had a matched control video that showed the same sequences of actions as the trick, but without VOE (e.g., following the same actions a ball would not change its color). Thus, we had a total of 18 magic videos (3 types of VOEs × 3 objects × 2 methods), with 18 matching control videos and nine unusual videos (3 unusual actions per used object). As the solutions to the magic tricks were revealed in distinct sets throughout the experiment, we used a variety of different methods for each type of VOE. This ensured that participants were only able to infer trick solutions they were intended to understand.

Prior to the fMRI experiment, we performed two psychophysics experiments with a total of 18 subjects (nine subjects in each experiment) to ensure the suitability of our stimuli and to select the magic tricks to be used in the fMRI experiment (see results in Supplementary Section*Behavioral evaluation of stimuli*).

#### 2.2.3 Luminance and durations

Tricks were recorded in a standardized setting under the same lighting conditions. To balance out remaining inequalities, custom MATLAB (MathWorks, Natick, MA) scripts were used to standardize the videos (resolution of 1920 x 1080 with 25 frames per second), which were filmed on different days and had different lengths. We manually applied white-balance using 5 to 10 selected white-pixels for all videos of a same day, matched the luminance and contrast of the videos based their first frame, and shortened the videos to be no longer than 14 s. The final duration of the videos was 12.8 s ± 1.08 s (mean ± SD).

#### 2.2.4 Stimulus presentation

Stimuli were presented using MATLAB 2019b using Psychtoolbox3 (version 3.0.16http://psychtoolbox.org/) on a Linux computer and back-projected to a translucent screen mounted at the rear of the scanner bore using a ProPixx projector (VPixx Technologies, Saint-Bruno-de-Montarville, Canada) at a frame rate of 144 Hz. Participants viewed the screen (26.1 x 14.7 visual degrees) via a mirror mounted on the 64-channel head coil (Siemens, Erlangen, Germany) at a distance of 105 cm. To center the stimulus presentation, we cropped 160 pixels from the left and right side of the videos that only showed a black background. Accordingly, the shown part of the videos covered 21.8 x 14.7 visual degrees (1600 x 1080 pixels).

### 2.3 Experimental design and procedure

#### 2.3.1 fMRI runs

Each run consisted of a total of 24 video trials: two repetitions of the six unique magic videos of one object (three types of VOE, each type recorded in two versions), resulting in 12 magic trick presentations; the six matching control videos (shown once); and two repetitions of three additional unusual videos, resulting in six unusual video presentations (seeFig. 1D). The resulting 24 video trials were presented in a pseudo-random order that avoided repetitions of identical videos (different randomizations across runs).

#### 2.3.2 Paradigm

The fMRI experiment consisted of three sets with four experimental fMRI runs each. Each set presented videos of only one object (i.e., balls, playing cards or pencils). The presentation order of the object sets was counterbalanced across subjects. After the first two fMRI runs in a set (pre-revelation runs), the method behind each magic trick in the set was revealed by showing the participants each of the magic tricks again together with the matching revelation video. Subjects could watch the videos as often as they wished and were asked to confirm per button press that they understood how each of the tricks was achieved. Thereafter, two more runs showing the same videos were performed (post-revelation runs). Together, each set consisted of two fMRI runs before and two fMRI runs after the explanation of the tricks. A visualization of the experimental design is shown inFigure 1E. We hypothesized that providing the explanation of the tricks would decrease VOE responses because participants would adjust their expectations (e.g., knowing where the seemingly disappearing objects were concealed). Consequently, our experimental design allowed us to compare responses with and without VOE using identical stimuli.

#### 2.3.3 Individual trials

Each video trial was presented for 14 s. In case a video was shorter (e.g., 13 s), the last frame of the video was shown for the remaining time (e.g., for 1 s). Participants were informed about this. Moreover, the first 500 ms of each video showed a central cue (1.3 visual degrees), indicating whether the video was going to be a magic (M) or a non-magic (X) video (i.e., control or unusual actions). We included the cue (M or X) at the beginning of the trial to prevent participants from being surprised by contextual or serial effects. Moreover, to reduce predictability of video content we flipped each video on every second presentation of the same video horizontally. After each video presentation, participants were asked to rate from 1 to 5 how surprising the content of the video was (1 = not surprising, 5 = very surprising). They had 2 s to respond, after which the next trial started (total trial duration = 16 s, seeFig. 1B). Behavioral surprise ratings were given by the subjects using a button-box with five keys.

#### 2.3.4 Pre-scan instructions

Before scanning, we instructed subjects about the task and design of the experiment (i.e., the set design, meaning of trial cues, explanation videos, etc.). Participants were informed that videos would show either magic tricks, control actions, or unusual actions. Moreover, participants were shown a magic and matching control example video (not used in the actual experiment) to give them an impression about the kind and duration of the videos they were about to see. Further, to prevent participants from watching the videos in a “problem-solving” attitude, we instructed them to passively watch and enjoy the videos without trying to get behind the method of the tricks, as these would be explained during the experiment.

### 2.4 fMRI data acquisition

fMRI data were acquired in a 3 Tesla Siemens Prisma scanner with a 64-channel head coil (Siemens, Erlangen, Germany). Functional images were acquired using an accelerated T2*-weighted gradient-echo echoplanar imaging (EPI) sequence (multiband factor = 2, repetition time (TR) = 2000 ms, echo time (TE) = 30 ms, flip angle (FA) = 75°, 62 slices with an isotropic voxel size of 2 × 2 × 2 mm) using GRAPPA (GRAPPA = 2). Each run consisted of 198 images (total duration = 6 min and 36 s). Moreover, a high-resolution T1-weighted structural scan with whole-brain coverage was performed for each participant (TR = 2000 ms, TE = 3.06 ms, inversion time (TI) = 1100 ms, FA = 9°, 192 slices and an isotropic voxel size of 1 × 1 × 1 mm). The structural scan was measured during the explanation of the tricks in the first set.

### 2.5 fMRI data preprocessing

Functional MRI images were preprocessed first by removing thermal noise from the magnitude EPI images using NORDIC, a PCA-based algorithm (Vizioli et al., 2021) in MATLAB. Then, we discarded the first five volumes of each run to allow for T1 equilibration effects. Using SPM12, we further performed motion correction (realigned to the first image), slice-time correction (using middle slices as reference), and co-registration to the structural scan. Finally, functional MRI data for whole-brain analyses were normalized to the Montreal Neurological Institute template brain (MNI152) and spatially smoothed with a Gaussian kernel of full width at half-maximum of 6 mm for univariate whole-brain analyses. Region-of-interest (ROI) analyses were performed on unsmoothed data in native space. Moreover, for the generation of subject-specific ROIs, we generated inflated individual brain surfaces using Freesurfer 7.1.1 (Dale et al., 1999) using a dedicated docker container (https://hub.docker.com/r/freesurfer/freesurfer).

### 2.6 Univariate whole-brain data analysis

To investigate differences in neural activity during high-level VOE in the human brain, we performed whole-brain and ROI univariate analyses. We had three specific aims: 1) to investigate neural responses to violations of physical principles in naturalistic stimuli, comparable to previous reports using magic tricks (cf.,Danek et al., 2015;Parris et al., 2009), 2) to investigate differential responses between different VOE (i.e., magic trick types), and 3) to investigate the role of prior knowledge in the perception of events violating physical principles by comparing responses to the very same videos before and after revelation.

For these analyses, we created two event-related general linear models (GLM) using the canonical hemodynamic response function and high-pass filtered data with a cut-off at 128s in SPM12. In the first GLM (aim 1), we modeled responses using only three regressors of interest in each run (magic, control, and unusual stimuli conditions). In a second, extended GLM, we investigated possible differential responses to different forms of violation of expectations (aim 2), using seven regressors of interest that modeled BOLD responses of the three magic types, three matching controls, and unusual actions for each run. As in previous reports, we modeled individual trials within a regressor using discrete event times based on the moment of surprise (cf.,Danek et al., 2015;Parris et al., 2009). The definition of the event times was done by averaging the independent selection of a suitable frame done by two authors (VP and PRG). For magic videos, timing was decided based on when the “magic” (i.e., VOE) happened (mean VOE onset was 7.62 s after video start; range: 4.36–9.64 s). For matching control videos, the corresponding time point was selected, that is, the moment in which one would expect to see a magic event in the magic videos (mean = 7.74 s; range: 4.52–10.92 s). For the videos showing unusual actions, timing was selected based on the onset of the unusual actions (mean = 6.06 s; range: 4.72–7.84 s). Moreover, we included participants’ response times, six movement parameters, and a column of ones as nuisance regressors in both GLMs. We additionally computed the same analyses modeling the whole video durations (14 s) and report these in theSupplementary Tables S11–S13andFig. S7). We used these models to address our three aims as follows.

Aim 1: to identify brain areas preferentially involved in signaling high-level VOE we compared responses to magic and matching control videos in all six pre-revelation runs (i.e., two runs from each object set) using the pooled regressors (from the first GLM) (*Magic_pre_*>*Control_pre_*). Similarly, we compared responses between the magic and the unusual videos (*Magic_pre_*>*Unusual_pre_*). First-level contrasts were used for second-level random-effect analyses using parametric tests as implemented in SPM12 and with non-parametric permutations tests using the non-parametric mapping toolbox SnPM13 (Nichols & Holmes, 2002). Moreover, we tested in significant clusters whether BOLD responses were positively correlated with subjective surprise ratings. We correlated parameter estimates of each video presentation taken from a separate GLM (seesection 2.9Multivariate pattern analysis MVPA), with the corresponding subjective surprise ratings after accounting for the video condition (partial correlation using the video condition as covariate), averaged the Fisher z-transformed correlation coefficients within a cluster, and tested for significance at group-level using one-sided T-tests against 0.

Aims 1 and 2: to investigate generic and violation-specific responses to high-level VOE before the explanation of the tricks, we used a second-level, 2 (magic, control) x 3 (appear, change, vanish) repeated-measures ANOVA to conduct four conjunction analyses (Friston et al., 2005). To find common responses to all VOE, we contrasted each magic trick type with its corresponding control condition before the explanation of the tricks (i.e.,*Magic_A_pre_*>*Control_A_pre_*) and used those contrasts to perform a conjunction (across all three VOE types). For each VOE type separately, we further performed a conjunction analysis on the responses of one VOE type against the others. For example, responses specific to an appearing object were investigated by means of the conjunction of the contrasts*Magic_A_pre_*>*Magic_C_pre_*∩*Magic_A_pre_*>*Magic_V_pre_*.

Aim 3: to identify brain areas generally involved in the perception of magic and affected by prior knowledge, we compared responses to magic after providing the explanation of the tricks*Magic_post_*>*Control_post_*and additionally compared the interaction between the revelation and magic (*Magic_pre_*>*Control_pre_*) > (*Magic_post_*>*Control_post_*). Finally, we compared the same contrasts for each magic type separately using the second, extended GLM, for example, (*Magic_A_pre_*>*Control_A_pre_*) > (*Magic_A_post_*>*Control_A_post_*). Please note that these contrasts are controlling for potential time confounds.

For all whole-brain analyses, a cluster-forming threshold of*p_unc_*= 0.001 and cluster size threshold of k = 30 voxels was applied. In tables, we report cluster-wise parametric and non-parametric FWE-corrected results as suggested byNichols and Holmes (2002). Clusters that survive FWE-correction are highlighted in bold. All non-parametric permutation tests were performed using 5000 permutations. Brain areas in whole-brain analyses were identified using the atlasreader toolbox (Notter et al., 2019), applying the Automated Anatomical Labeling atlas 3 (AAL3) (Rolls et al., 2020).

### 2.7 Univariate whole-brain control analyses

We performed two control analyses for the whole-brain univariate results. First, it is possible that the conjunction analyses examining differences between the magic trick types are confounded by differences in the visual content of the videos at specific time-points. This is because we are not only comparing videos showing “appearances”, “color changes”, and “vanishes”, but also videos showing “red objects”, “blue objects”, and “no objects” at a specific time point, respectively (seeFig. 1C). To control for this possible stimulus-driven confound, we compared responses between magic and control videos showing similar visual contents at specific time points. Second, our knowledge-dependent analysis could be confounded by general condition-independent time effects and, as participants were repeatedly presented the video stimuli within a set, by the repetition suppression effect (Grill-Spector et al., 2006;Krekelberg et al., 2006). To address these potential confounders, we performed a mixed-effects model, modeling the change in prior-knowledge, experiment-wise and set-wise temporal decays, to explain the average beta estimates of the magic tricks of each run for each significant cluster of the prior-knowledge dependent contrast (*Magic_pre_*>*Control_pre_*) > (*Magic_post_*>*Control_post_*).

Detailed methods of our control analyses can be found in Supplementary Sections*VOE-specific control analysis*and*Prior knowledge dependent control analysis*respectively.

### 2.8 ROI definition

For the ROI analyses, we defined 26 hypothesis-driven ROIs, separated into two groups.

First, we defined a set of 16 surprise-related ROIs based on significant responses to magic videos from previous experiments (Danek et al., 2015;Parris et al., 2009). We defined 14 frontal and parietal ROIs by combining labels from a multi-modal parcellation of the human cortex (Glasser et al., 2016) and two subcortical ROIs using the Freesurfer automatic parcellation (Fischl et al., 2002).

Second, we used a probabilistic map of visual fields (Wang et al., 2015) to define 10 visual ROIs: primary visual cortex (V1), secondary visual cortex (V2), V3, V3A, V3B, human V4 (hV4), lateral occipital and ventral complex (LO and VO, respectively), intraparietal sulcus (IPS), and frontal eye-fields (FEF). All ROIs were defined in native space. For a detailed list of ROIs, please see Supplementary Section*Region of interest definition*.

We included these visual ROIs to use in the decoding of the VOE type (i.e., appear, change and vanishing, see below) and to test for differences evoked by the different VOE types and the effect of prior knowledge. For example, areas of the ventral visual cortex are known to be responsive to color (Bartels & Zeki, 2000), while the lateral occipital complex is responsive to objects (Grill-Spector et al., 2001). As predictive coding approaches predict feature-specific prediction errors in functionally specialized regions, we expect unexpected color changes to affect color-responsive ROIs (e.g., hV4, VO and PH), and unexpected object appearances to affect object-responsive areas LO and VO, in line with recent imaging evidence (Jiang et al., 2016;Richter et al., 2018;Stefanics et al., 2019). Early visual areas (V1, V2, V3) were included to investigate possible top-down effects of prior knowledge in lower-level areas, when comparing the exact same videos before and after revelations, while the parietal (IPS) and prefrontal ROIs (FEF) were included due to their involvement in top-down voluntary attention (Corbetta & Shulman, 2002). SeeFigure 5Afor a depiction of all 26 ROIs in an exemplary subject.

### 2.9 Multivariate pattern analysis (MVPA)

Apart from the univariate analyses testing for net signal differences, we further wanted to investigate which areas of the brain carry pattern information about the different types of VOE (unexpected appearance, feature change, and omission). To do so, we performed a series of multivariate pattern analyses (MVPA) on the 26 hypothesis-driven ROIs and a control ROI.

For the decoding analyses we computed a GLM in which every trial (i.e., video presentation) was modeled as a separate regressor to increase the number of data points for training and testing. All analyses were performed using a shrinkage linear discriminant analysis (LDA) on the de-meaned beta estimates of the individual trials (by the mean over all estimates, i.e., all trials, within each voxel) using the Python (version 3.8.13) package scikit-learn’s class LinearDiscriminantAnalysis (Pedregosa, 2011). To examine if any of the ROIs contained information about the different types of VOE (appear, color change, and vanish), we trained and tested our decoder to predict the VOE types following a three-fold cross-validation scheme to ensure generalization across objects. We trained on the data of two objects (i.e., estimates from two sets, 48 trials) and tested on the third object (i.e., estimates from the third set, 24 trials). Significance testing of the decoding accuracies was done using a permutation analysis (1000 permutations) implementing the max statistic correction to correct for multiple comparisons (Nichols & Holmes, 2002). A control ROI (third ventricle) was included in the analysis, which should carry no information and thus reflect chance level.

Decoding analyses were performed separately for data before and after explanations of the magic tricks. Permutation-based corrected significance thresholds were 36.98% and 36.92% before revelation and after revelation, respectively. As both analyses were conducted using estimates based on the very same videos, we hypothesized that any significant difference in decoding accuracies between data before and after revelation would be indicative of decodable prior-knowledge dependent surprise signals. We tested for differences in decoding accuracies using paired t-tests between decoding using pre-revelation data and decoding using post-revelation data, only in those ROIs that showed significant decoding (corrected) using pre-revelation data. Using the same ROIs, we performed the same decoding analyses but using beta estimates from GLMs that modeled the moments 5 s before and after the onset VOE in steps of 2 s (i.e., -5, -3, -1, 1, 3, 5 s relative to VOE onset). We expect decoding accuracies to peak around VOE onset, because differences in signals should be strongest, and decrease the further we move away from VOE onset.

Additionally, as an exploratory approach we performed a similar whole-brain searchlight analysis on unsmoothed data in MNI-space using a sphere to decode the magic types (4 mm radius), separately for data before and after explanation of the tricks using the SearchLight class implemented in nilearn (Abraham et al., 2014). The searchlight whole-brain accuracy maps were spatially smoothed with a 4 mm Gaussian kernel. A permutation-bootstrap hybrid method (in which each randomly generated accuracy map was also smoothed with a 4 mm Gaussian kernel) was used for significance testing and correction for multiple comparisons (Stelzer et al., 2013) using custom-made Python code.

### 2.10 Behavioral data

To test for differences in surprise ratings between videos before and after the explanation of the tricks, we performed a 2 (before/after explanation) x 3 (magic, unusual and control videos) repeated-measures ANOVA. We expected to see higher surprise ratings for magic videos compared to control videos and higher ratings for magic videos before compared to after the revelation of the methods. We also wanted to test if videos of magic and of unusual actions led to similarly high surprise ratings. Moreover, we tested for differences in surprise ratings of the magic videos for different objects and VOE types in 2 x 3 repeated-measures ANOVAs (rmANOVA) with the factors revelation (before/after) and object (ball/card/pencil) or VOE type (appear/change/vanish), respectively.

### 2.11 Eye tracking

Gaze positions were measured using an MR-compatible Eyelink 1000 (SR-Research, Ottawa, Canada) positioned at the rear end of the scanner bore. Eye tracking data were analyzed to test whether gaze positions, number of blinks, and number of saccades differ significantly between conditions. Details about data acquisition, preprocessing, and analyses can be found in Supplementary Section*Eye tracking acquisition and analysis*.

### 2.12 Inference statistics

Effect sizes for repeated-measures ANOVAs and paired tests are presented as partial eta squared (*η*^2^) and Cohen’s*d*, respectively. Sphericity of rmANOVAs was tested using the Mauchly test. If sphericity was violated, degrees of freedom were adjusted using the Greenhouse-Geisser correction, the corresponding ε-correction factor is provided. Normality of data was assessed using the Shapiro-Wilk test (for paired tests and post-hoc tests). In case that data were normally distributed, we performed paired t-tests, otherwise we performed non-parametric Wilcoxon signed-rank tests instead. Correction for multiple comparison was performed using a step-down Holm-Bonferroni correction. Please note that in the post-hoc tests we corrected for each hypothesis separately (i.e., p-values for post-hoc tests of one factor are corrected independent of another factor or an interaction). In general, corrected p-values (*p_corr_*) are reported in text, uncorrected p-values (*p_unc_*) are reported in the corresponding tables. The threshold for statistical significance was set to 0.05 for all tests.

## 3 Results

### 3.1 Behavioral surprise ratings

Behavioral data were first tested for differences in surprise ratings for video condition (magic, control, and unusual videos) and revelation condition (before and after revelation) using a 3 × 2 rmANOVA (seeFig. 2A). In sum, this analysis revealed that magic tricks were perceived as more surprising than the control videos, and that surprise ratings dropped after explanation of the tricks. In detail: the analysis revealed significant main effects for both factors (video condition:*F*(2,46) = 57.494,*p_unc_*< 0.001,*η*^2^= 0.478, ε = 0.932, revelation:*F*(1,23) = 103.242,*p_unc_*< 0.001,*η*^2^= 0.211, ε = 1) and interaction:*F*(2,46) = 43.081,*p_unc_*< 0.001,*η*^2^= 0.134, ε = 0.641). As expected, post-hoc Wilcoxon signed-rank tests revealed that magic videos were more surprising than control and unusual videos, before (*W*= 1,*p_corr_*< 0.001, Cohen’s*d*= 4.07 and*W*= 8,*p_corr_*< 0.001, Cohen’s*d*= 2.06, respectively) and after explanation of the tricks (*W*= 5,*p_corr_*< 0.001, Cohen’s*d*= 1.55 and*W*= 26,*p_corr_*< 0.001, Cohen’s*d*= 0.812, respectively), and that all video conditions were more surprising before compared to after the revelation (all three video conditions:*p_corr_<*= 0.001). Unusual videos were more surprising than control videos pooled across runs (*W*= 42,*p_corr_*= 0.006, Cohen’s*d*= 0.59), but significantly so only in the first two runs (*W*= 26,*p_corr_*= 0.002, Cohen’s*d*= 0.71) (seeSupplementary Table S1for a detailed report of all post-hoc tests).

**Fig. 2.**
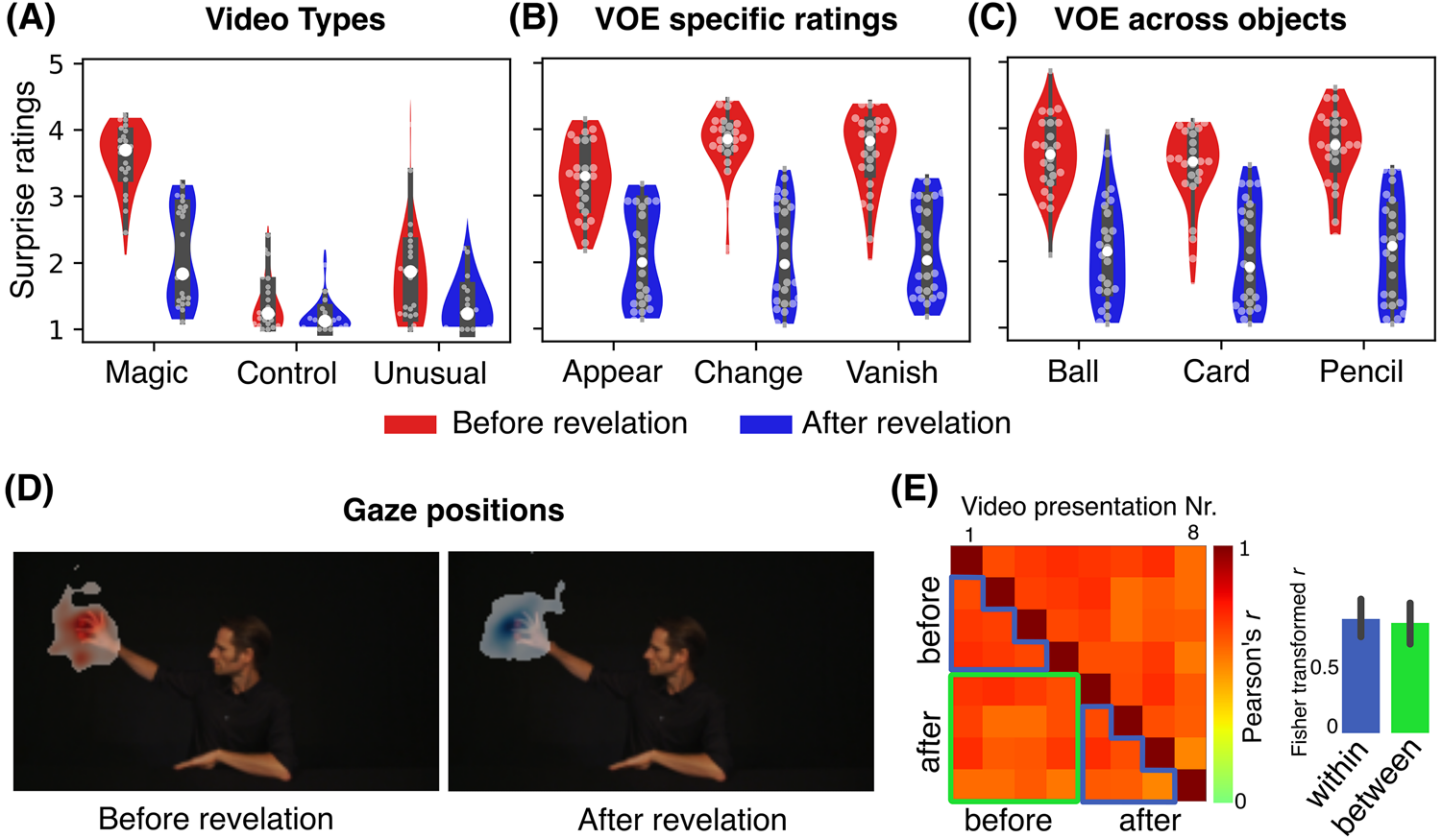
Shown are behavioral surprise ratings separated for the different video types (A), for magic videos across VOE types (B), and across objects (C). Surprise ratings before the revelations of the magic tricks (red) were consistently higher than after the revelations (blue). (D), Exemplary group gaze positions for the same magic video in the moment a ball appears, before (left) and after (right) the revelation of the trick. (E), Correlation matrix for gaze positions around the moment of magic (-1 s and +2 s) from an exemplary video. To test for differences between gaze paths before and after the revelation of the tricks, we compared (paired t-tests) the average Fisher z-transformed correlations between gaze paths of all combinations of presentations (from presentation Nr. 1 to Nr. 8)*between*pre- and post-presentations (marked green) and those*within*pre- and*within*post-presentations (marked blue), as shown in the bar plot.

Average surprise ratings of magic videos were consistently high before the explanation of the tricks (all group means > 3) and decreased afterward (all group means < 2.5) (seeFig. 2A, B and C). We further performed two rmANOVAs using only ratings from magic videos, to test for possible differences in VOE types and the objects used. Both rmANOVAs included the revelation condition as a factor and showed a significant decrease in surprise rating after the revelation of the tricks (as expected from the previous analysis). We additionally found a significant main effect for the magic type (*F*(2,46) = 13.8,*p_unc_*< 0.001,*η*^2^= 0.032, ε = 0.848) and an interaction of magic type and revelation condition (*F*(2,46) = 11.02,*p_unc_*< 0.001,*η*^2^= 0.018, ε = 0.795) (seeFig. 2B). Post-hoc tests showed that appearances were rated less surprising than color changes and disappearances pre-revelation (*W*= 12,*p_corr_*< 0.001, Cohen’s*d*= -0.835 and*W*= 35.5,*p_corr_*= 0.002, Cohen’s*d*= -0.631, respectively). No difference between color change and vanish magic tricks was observed (seeSupplementary Table S2). No main effect for objects nor an interaction of object and revelation condition were found (all*F-values*< 2, all*p_unc_*> 0.15 and*η*^2^< 0.012) (seeFig. 2C).

### 3.2 Eye-tracking results

Eye-tracking data were tested for systematic differences in gaze traces, saccades, and blinks during viewing of the videos (see also Supplementary Section*Eye-tracking results*andFig. S2). Gaze traces between pre- and post-revelation runs were similar for all videos (see an example visualization inFig. 2D) and no significant difference of correlations of gaze traces was observed (all*p_unc_*> 0.2) (seeFig. 2Efor an example). Moreover, the number of saccades and blinks around the VOE times were similar between experimental conditions and only revealed small differences we deem unlikely to have affected the imaging results.

### 3.3 Univariate whole-brain analysis

#### 3.3.1 Whole-brain surprise responses

To investigate neural correlates of high-level VOE when viewing seemingly impossible events, we first compared whole-brain responses to the magic and matched control videos before the revelation of the magic tricks (*Magic_pre_*>*Control_pre_*). This contrast revealed several clusters of activity in frontal and parietal cortices, largely in line with previous studies (Danek et al., 2015;Parris et al., 2009) (seeFig. 3AandTable 1). In particular, large clusters of activity were observed in the medial part of Brodmann area 8 (preSMA), the dorsal and ventral anterior cingulate cortex (dACC and vACC), and the posterior parietal cortex (PPC, especially the superior parietal lobe and the precuneus). Moreover, lowering the cluster size threshold to k = 10 revealed subcortical areas such as left caudate nucleus (k = 19), in line with previous studies (Danek et al., 2015;Parris et al., 2009) and bilateral Thalamus (left k = 22, right k = 13). No lower-level sensory area was differentially modulated in view of the unexpected events. Partial correlations between beta estimates of each magic or control video presentation and corresponding surprise ratings (using video condition as covariate) showed significant positive correlations in the dACC/preSMA, parietal cortex (including PPC and bilateral anterior IPS) and left and right SFG (all T-values > 1.78, all p-values < 0.044).

**Fig. 3.**
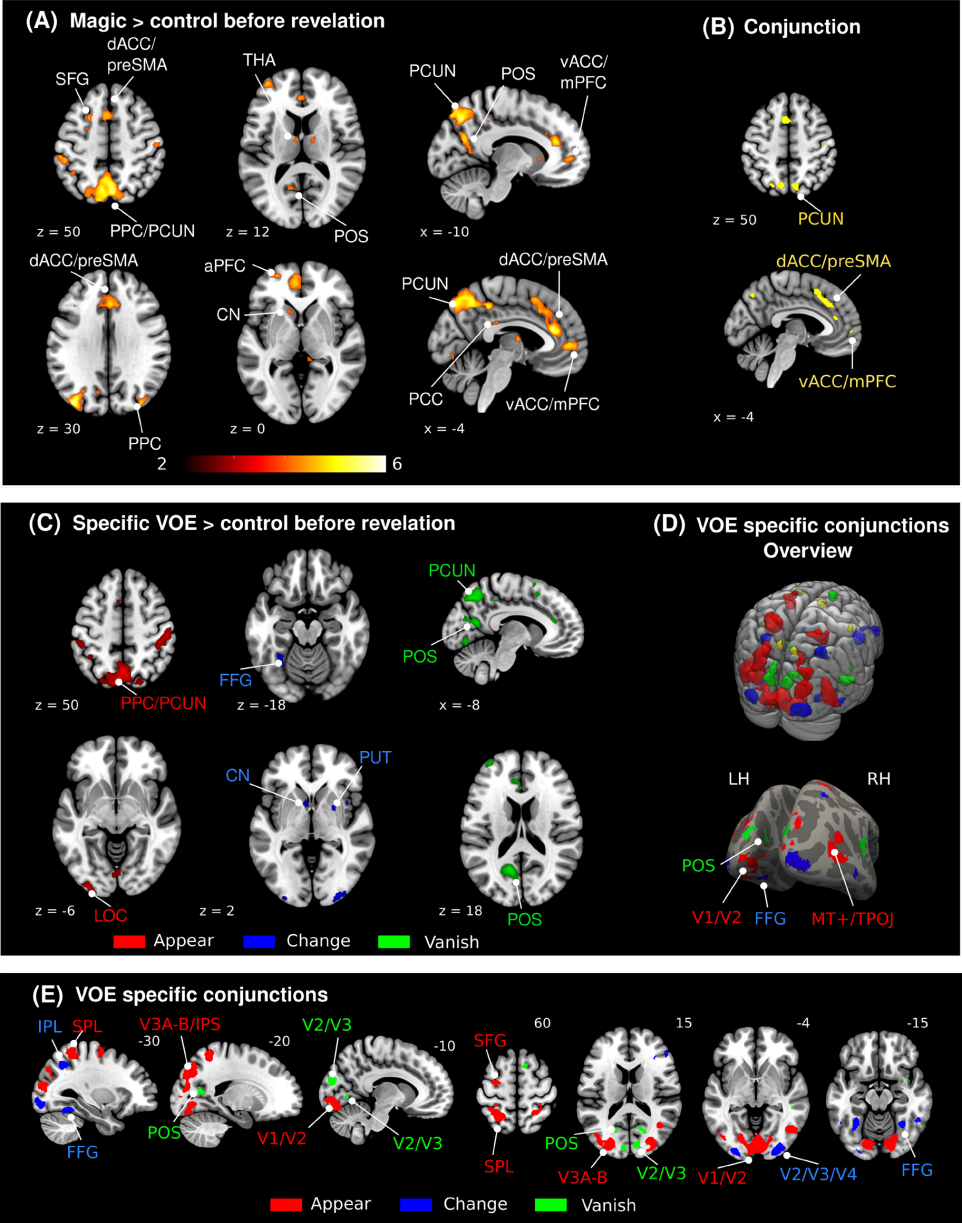
(A), shown are active regions during viewing magic tricks compared to matched control videos before the participants knew how the tricks were performed (thresholded at*p_unc_*< 0.001 and k =30, uncorrected). (B), shown are results from the conjunction analyses testing for generic activity (left, yellow) (at*p_unc_*< 0.005). Only the dorsal ACC and preSMA and the precuneus (PCUN) revealed generic responses to high-level VOE at*p_unc_*< 0.001. (C), VOE-specific activity revealing several posterior visual areas responsive to appearances (red), color changes (blue), and objects disappearing (green). (D), overview of all conjunctions results in the MNI152 template volume (upper) and of the VOE-specific responses projected on an average surface (fsaverage) (lower). (E), shown are VOE-specific conjunction results. ACC: anterior cingulate cortex; dACC: dorsal ACC; vACC: ventral ACC; CN: caudate nucleus; aPFC: anterior prefrontal cortex; FFG: fusiform gyrus; IPL: interior parietal lobe; IPS: intraparietal sulcus; MT+: motion area MT; mPFC: ventral prefrontal cortex; PCC: posterior cingulate cortex; PCUN: precuneus; POS: parieto-occipital sulcus; PPC: posterior parietal cortex; SFG: superior frontal gyrus; SMA: supplementary motor area; SPL: superior parietal lobe; THA: thalamus; TPOJ: temporo-parietal-occipital junction. LH: left hemisphere; RH: right hemisphere.

**Table 1.** Significant clusters of activity from the whole-brain contrast comparing responses between magic videos and matched controls (*Magic_pre_*>*Control_pre_*, thresholded at p ≤ 0.001 and k = 30, uncorrected) and the conjunction analysis testing for common areas involved in the processing of violation of expectations (thresholded at*p_unc_*≤ 0.001 and k = 30, uncorrected).

**Brain region**	**AAL atlas labels**	**p(FWE) permutation**	**p(FWE) parametric**	**p(unc)**	**k**	**T**	**x**	**y**	**z**
** Magic _pre_** **>** ** Control _pre_**									
**Posterior parietal cortex**	**Occipital_Mid_L**	**0.0004**	**<0.001**	<0.0001	1871	7.16	-34	-82	34
	**Precuneus_R**	-	-	-	-	6.87	4	-68	52
	**Precuneus_L**	-	-	-	-	6.48	-10	-68	44
**daCc**	**Cingulate_Ant_L**	**0.0048**	**<0.001-**	0.0005	886	6.46	-6	30	20
	**Cingulate_Ant_R**	-	-	-	-	6.05	4	32	24
	**Cingulate_Mid_R**	-	-	-	-	5.51	2	24	32
**L. Parieto-occipital sulcus**	**Cuneus_L**	0.0594	**0.016**	0.0082	162	6.18	-14	-62	22
	**Calcarine_L**	-	-	-	-	4.21	-12	-60	14
**R. Intraparietal sulcus**	**Occipital_Mid_R**	0.1752	0.230	0.0284	74	5.53	36	-80	32
**L. anterior PFC**	**Frontal_Sup_2_L**	0.1982	0.288	0.0332	67	5.41	-26	58	4
**L. anterior intraparietal**	**Postcentral_L**	0.0548	**0.012**	0.0074	172	5.2	-46	-36	54
	**Parietal_Inf_L**	-	-	-	-	4.47	-42	-38	44
**vACC/mPFC**	**Cingulate_Ant_L**	0.0548	**0.012**	0.0074	172	5.13	-4	48	2
**L. Superior frontal gyrus**	**Frontal_Sup_2_L**	0.0522	**0.010**	0.007	179	5.02	-22	12	58
	**Frontal_Sup_2_L**	-	-	-	-	3.7	-22	0	52
**L. anterior middle frontal gyrus**	**Frontal_Mid_2_L**	0.1722	0.223	0.028	75	5.01	-34	54	14
**L. Postcentral gyrus**	**Postcentral_R**	0.1134	0.091	0.0173	103	4.71	52	-16	54
	**Postcentral_R**	-	-	-	-	4.07	52	-18	46
	**Postcentral_R**	-	-	-	-	3.72	46	-22	42
**R. Superior frontal gyrus**	**Frontal_Sup_2_R**	0.2394	0.395	0.0417	57	4.61	28	10	62
**L. PCC**	**no_label**	0.2832	0.503	0.0511	49	4.48	-2	-30	26
**Conjunction of** *** Magic _pre_*** **>** *** Control _pre_*** **per VOE type**									
**dACC/preSMA**	**Supp_Motor_Area _L**	n.a.	0.722	0.096	38	4.02	-4	16	50
**Precuneus**									
	**Precuneus_R**	n.a.	0.722	0.096	38	3.94	8	-68	48

p(FWE) permutation shows permutation-based cluster statistics. p(FWE) parametric shows parametric cluster statistic. Clusters that survive FWE-correction are highlighted in bold. k = cluster size,*T*= t statistic at peak voxel, x, y, z = peak voxel MNI coordinates [mm]. dAAC: dorsal anterior cingulate cortex; PFC: prefrontal cortex; vACC: ventral ACC; mPFC: medial PFC; PCC: posterior cingulate cortex; DLPFC: dorso-lateral prefrontal cortex; preSMA: supplementary motor area.

When comparing whole-brain responses to the magic and the unusual videos before the revelation of the magic tricks (*Magic_pre_*>*Unusual_pre_*), similar frontal (dACC) and parietal areas (PPC) were active (seeSupplementary Fig. S3and Tables S3–S6). Additionally, the right anterior insula, as well as wide-spread occipital and parietal sensory areas (e.g., V1–V3, LO1–2 and IPS) showed increased activity during the presentation of magic videos compared to the videos showing unusual actions. However, a similar pattern of results was also observed when comparing control videos to unusual action videos (*Control*>*Unusual*) (seeSupplementary Fig. S3A–BandTables S3–S6), suggesting that the responses observed may not be solely driven by surprise, but by other factors instead (e.g., differences in visual content).

Inverting the magic related contrasts (i.e.,*Control_pre_*>*Magic_pre_*and*Unusual_pre_*>*Magic_pre_*) revealed, among others, engagement of temporal sulcus (STS) extending into the temporo-parietal junction (TPJ) and the ventro-medial prefrontal cortex (vmPFC) (seeSupplementary Fig. S3C), which are areas known to be involved in social cognition (Deen et al., 2015;Hiser & Koenigs, 2018;Lahnakoski et al., 2012;Saxe & Kanwisher, 2003).

#### 3.3.2 Generic surprise responses

To specifically test for generic surprise responses in the brain showing a significant involvement of all three types of VOE, we performed a conjunction analysis combining all different VOE types before the revelation of the tricks (*Magic_A_pre_*>*Control_A_pre_*∩*Magic_C_pre_*>*Control_C_pre_*∩*Magic_V_pre_*>*Control_V_pre_*). We found clusters in the dorsal anterior cingulate cortex (dACC) bilaterally and right posterior parietal cortex (precuneus) revealing generic responses (seeFig. 3B, left andTable 1). Also, using a more liberal threshold of*p_unc_*≤ 0.005 (and k = 10), we further observed generic activity in the vACC/mPFC and the left precuneus.

#### 3.3.3 Specific surprise responses

After establishing what areas are generally involved in the processing of violation of expectations (i.e., commonly active in seemingly impossible appearances, disappearances, and color changes), we looked for VOE type-specific differential activity in the brain (e.g., areas responsive to something unexpected appearing but not disappearing or changing color). Beyond the systematic generic activation of frontal and parietal areas described above, all three types of VOE evoked responses in posterior visual areas (seeFig. 3CandSupplementary Table S8). Activations specific to each type of VOE were largely confined to posterior sensory areas: appearances induced an increase in activity in V1 and the intraparietal sulcus, color changes in areas of the inferior temporal cortex and ventromedial visual cortex and vanishes in the parieto-occipital sulcus. To better visualize this diverse modulation of sensory areas by the different VOE, we tested which areas were significantly more activated by one type of VOE than by the other two using a conjunction test (e.g., test for appear-specific responses:*Magic_A_pre_*>*Magic_C_pre_*∩*Magic_A_pre_*>*Magic_V_pre_*) (seeFig. 3D–EandSupplementary Table S7). Activity related to objects appearing was observed in more medial parts of early visual areas (peak coordinates: x = 30, y = -88, z = 22), in higher-level visual areas of the lateral and middle occipito-temporal cortex (peak coordinates: x = 46, y = -66, z = 2) and bilateral intraparietal sulcus (peak coordinates: x = 30, y = -88, z = 22 and x = -22, y = -78, z = 44), while activity related to color changes was observed specifically in more posterior parts of secondary visual areas (V2/V3/V4, peak coordinates: x = ±30, y = -96, z = -6), bilaterally in the IPS (peak coordinates: left IPS x = -28, y = -60, z = 4 and right IPS x = 32, y = -54, z = 44) and in color-responsive ventral areas of the fusiform gyrus (FFG, peak coordinates: x = -30, y = -50, z = -16 and x = 32, y = -54, z = -14). Finally, vanishing objects evoked significant responses in the anterior parts of the calcarine sulcus, close to the parieto-occipital sulcus (peak coordinates: x = -20, y = -64, z = 12 and x = 18, y = -76, z = 8) and in the right superior temporal sulcus (STS, peak coordinates: x = 54, y = -38, z = 6).

##### 3.3.3.1 Control analysis for visual content

As these VOE type-specific patterns of activity are located predominantly in visual processing areas, they are potentially related to general visual differences between the conditions tested. The compared videos are not only showing “appearances”, “color changes”, and “vanishes”, but also “red objects”, “blue objects”, or “no objects”, respectively.

To test if these VOE type-specific responses are confounded by different visual input, we performed a control analysis comparing neural responses between 1) videos showing red objects, either as the product of a magic trick (appearances) or as a control to other tricks (vanishes) (*Magic_A_pre_*>*Control_V_pre_*) and 2) between videos showing no object, either as the product of a magic trick (vanishes) or as a control to the other tricks (appearances) (*Magic_V_pre_*>*Control_A_pre_*). Differential responses in posterior visual areas to these control contrasts were similar to the results of the corresponding conjunction analyses (for appearances and vanishes), suggesting that violation-specific responses observed in posterior visual areas are unlikely to be driven merely by visual content (see overlays inSupplementary Fig. S5). However, if these signals in visual areas reflect specific prediction errors based on different VOE, we would additionally expect them to be modulated by prior knowledge and to show decreased responses after the explanation of the tricks.

#### 3.3.4 Prior-knowledge dependent whole-brain responses

To investigate the effect of prior knowledge on brain responses, we provided participants with the methods behind the magic tricks. Surprisingly, neural activity after explanation of the tricks (*Magic_post_*>*Control_post_*) was similar to that before the explanation of the tricks (seeFig. 4AandSupplementary Table S10). Thus, areas related to the processing of surprising events remained significantly active in view of VOE also after the explanation of the tricks (i.e., dACC, caudate nucleus, anterior insula). The interaction contrast comparing surprise responses before and after providing the explanations (*Magic_pre_*>*Control_pre_*) > (*Magic_post_*>*Control_post_*) revealed only a small number of areas showing prior-knowledge dependent modulations (seeFig. 4B–CandTable 2). We observed higher activation in a large cluster of the medial prefrontal cortex (mPFC) and ventral ACC (peak coordinates: x = 2, y = 46, z = -10) and right posterior cingulate cortex (PCC) with FWE-correction (peak coordinates: x = 6, y = -46, z = 8). These regions, hence, decreased magic-related activity after the revelation. These areas overlap with and constitute a subset of the activity observed with*Magic_pre_*>*Control_pre_*before the revelation of the tricks.

**Fig. 4.**
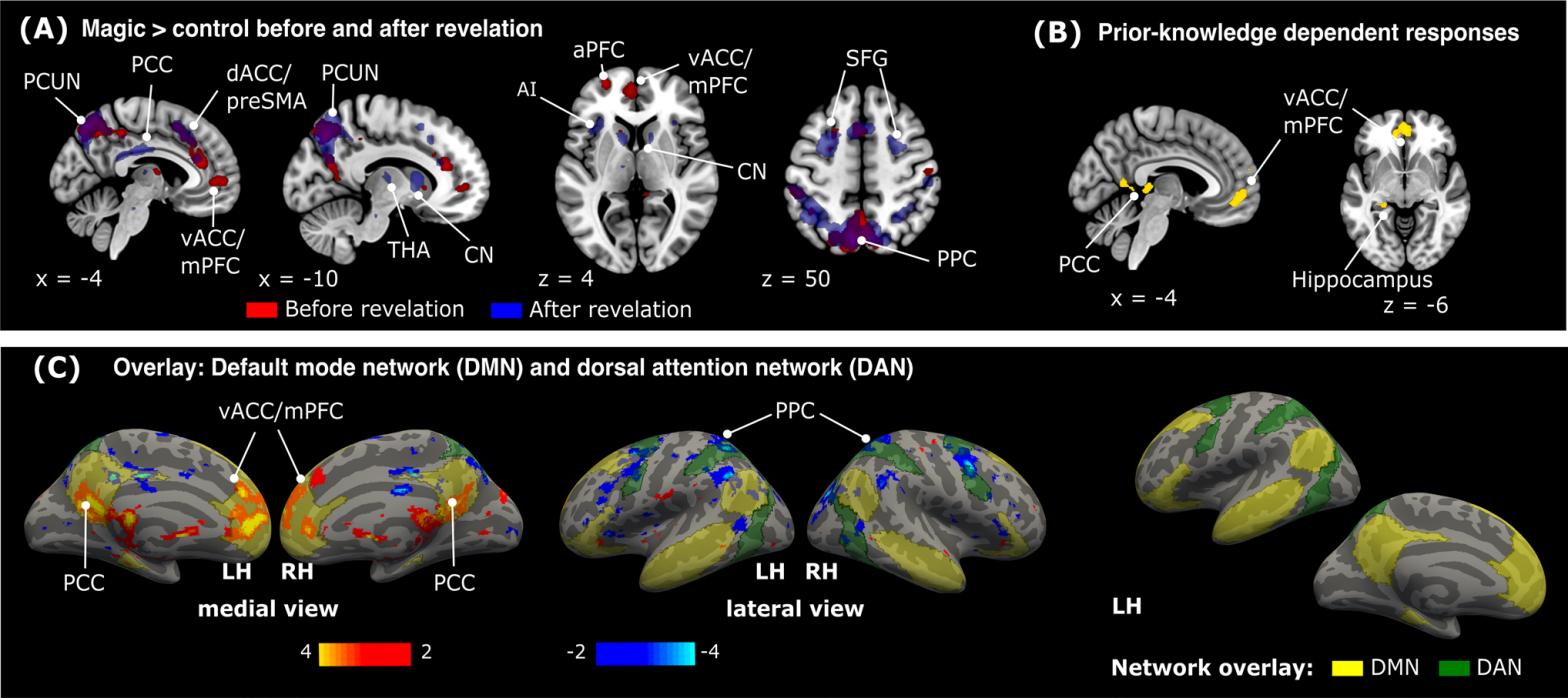
Prior knowledge dependent modulation of brain responses. (A), overlay of*Magic*>*Control*before (red) and after (blue) explanation of the magic tricks. Both contrasts reveal a consistent activation of surprise related areas, such as the dorsal ACC (dACC), caudate nucleus (CN), and posterior parietal cortex (PPC). (B), the difference of both contrasts (i.e., the interaction between video condition and revelation) revealed only few areas that were significantly more active before the explanation of the tricks: the posterior cingulate cortex (PCC), the ventral ACC/medial prefrontal cortex (vACC/mPFC), and left Hippocampus. Threshold at*p_unc_*< 0.001 and k = 30. (C), shown are the interaction testing for prior-knowledge dependent responses (thresholded from*t*= 2 to*t*= 4, red to yellow and*t*= -2 to*t*= -4, dark blue to light blue) and the default mode network (DMN) and the dorsal attention network (DAN) as transparent overlay projected on to an average brain surface (fsaverage). The network overlays are provided byYeo et al. (2011). AI: anterior insula; aPFC: anterior prefrontal cortex; LH: left hemisphere; PCUN: precuneus; preSMA: supplementary motor area; RH: right hemisphere; SFG: superior frontal gyrus; THA: thalamus.

**Table 2.** Results of contrasts comparing neural responses to VOEs before and after explanation of the tricks (thresholded at*p_unc_*< 0.001 and k = 30, uncorrected).

**Brain region**	**AAL atlas labels**	**p(FWE) permutation**	**P(FWE) parametric**	**p(unc)**	**k**	**T**	**x**	**y**	**z**
**vACC/mPFC**	**Frontal_Med_Orb_R**	**0.0068**	**<0.001**	0.0007	538	5.72	2	46	-10
	**Frontal_Med_Orb_L**	-	-	-	-	4.96	-10	42	-6
**R. Cuneus (V3)**	**Cuneus_R**	0.25	0.466	0.0385	52	5.23	8	-86	34
**L. POS**	**Precuneus_L**	0.1082	0.100	0.014	101	4.99	-8	-56	8
**L. PCC**	**Calcarine_L**	-	-	-	-	4.31	-6	-48	4
**R. PCC**	**Precuneus_R**	**0.0424**	**0.009**	0.005	185	4.91	6	-46	8
	**no_label**	-	-	-	-	4.59	-4	-30	6
	**no_label**	-	-	-	-	4.56	8	-36	4

p(FWE) permutation shows permutation-based cluster statistics. p(FWE) parametric shows parametric cluster statistic. Clusters that survive FWE-correction are highlighted in bold. k = cluster size, T = t statistic at peak voxel, x, y, z = peak voxel MNI coordinates [mm]. ACC: anterior cingulate cortex; vACC: ventral ACC; mPFC: medial prefrontal cortex; POS: parieto-occipital sulcus; PCC: posterior cingulate cortex; dACC: dorsal ACC.

Interestingly, the observed decrease of activity in the vACC/mPFC and PCC after revelation of the tricks coincides with the midline core areas of the default mode network (DMN), while parietal areas of the dorsal attention network (DAN) showed increased activity. This indicates that our findings are unlikely a result of a decrease in attention, as the DAN is known to direct top-down attention (Corbetta & Shulman, 2002). A visualization of these patterns of activity, together with the DMN and DAN is shown inFigure 4C.

##### 3.3.4.1 No prior-knowledge dependency of trick-specific modulations

While prior-knowledge driven signals overlapped with neural activations to unspecific VOE (*Magic_pre_*>*Control_pre_*), none of the previously observed trick-specific visual areas showed modulation as a factor of prior knowledge. Further, the contrasts investigating the effect of prior knowledge for each VOE type separately revealed similar response patterns to those reported in the main contrasts (seeSupplementary Fig. S4). This indicates that visual areas responsive to a specific VOE (e.g., an object changing color) were not modulated by prior knowledge.

##### 3.3.4.2 Controlling for time effects in net responses

To rule out possible time confounds in results comparing responses before and after revelation of the tricks, we inspected how the individual values changed across time as a control analysis by means of a mixed-effects model. Consistent with responses being driven by prior-knowledge, a pre-post predictor was significant in all clusters (all*p_corr_*< 0.05). However, in one cluster (right Cuneus, x = 8, y = -86, z = 34; k = 52) a significant amount of variance was also explained by the time-decay regressor modeling the time of the experiment, showing that the activity in that cluster had a contribution of time. Yet, the fact that in all clusters the pre-post regressor remained significant despite inclusion of the time-regressor shows that knowledge-dependent effects were true (seeSupplementary Fig. S6andTable S9).

### 3.4 Univariate ROI analysis

To complement the whole-brain analysis, we defined a set of 26 hypothesis-driven regions-of-interest (ROIs) from which we extracted and analyzed parameter estimates and tested them with paired t-tests (correcting for the number of ROIs). Overall, our ROI-based analyses confirmed our above whole-brain findings. None of the lower-level visual cortices showed a significant increase of neural responses during the magic compared to the matched control condition before the explanation of the tricks (all*p_unc_*> 0.24). In contrast, significant responses to magic were observed in the visual parietal ROI IPS (*t*(23) = 2.746,*p_unc_*= 0.012, Cohen’s*d*= 0.53), as well as in several higher-level surprise-related ROIs, especially in adACC (*t*(23) = 5.32*, p_corr_*= 0.001, Cohen’s*d*= 0.917), as well as in vACC, pvACC, BA6, BA46, and caudate nucleus (all*p_unc_*< 0.05, see detailed results inSupplementary Tables S14–S17). Only two ACC ROIs (adACC and vACC) and 8BM (directly superior to the adACC) showed a decrease of activity after the revelation of the tricks (*t*(23) = 3.43,*p_unc_*= 0.002, Cohen’s*d*= 0.41,*W*= 79,*p_unc_*= 0.042, Cohen’s*d*= 0.42 and*t*(23) = 2.54,*p_unc_*= 0.01, Cohen’s*d*= 0.41, respectively). None of the visual ROIs showed a similar response pattern. In contrast, VOE-specific responses were largely constrained to visual ROIs (see Supplementary Section*Univariate ROI results*).

### 3.5 Multivariate pattern analysis

Our univariate analyses revealed generic responses (modulated by prior knowledge) in frontal and parietal areas, while showing trick-specific responses in posterior sensory areas (unaffected by prior knowledge). However, differences in specific VOE and prior-knowledge modulations might also be reflected in activation patterns and not only in net signal differences. Accordingly, we complemented our univariate analysis by a multivariate pattern analysis to investigate if specific information about VOE types were present in the activity patterns of our surprise-related frontal and parietal ROIs (which showed only generic responses to magic).

#### 3.5.1 Visual ROIs

Decoding of the specific VOE types across objects was possible in all posterior visual ROIs (V1, V2, V3, hV4, V3A, V3B, LO, VO, IPS) before the explanation of the magic tricks (all corrected p-values < 0.001; corrected using a permutation maximal statistic,Nichols & Holmes, 2002). After revelation of the method behind the magic tricks, decoding accuracies significantly dropped in most ROIs (V1, V2, V3, LO, and IPS) (all*p_corr_*<= 0.05), being below threshold in LO and IPS (seeFig. 5BandSupplementary Table S20). Additionally, we found uncorrected significant differences in hV4 and V3B (*p_unc_*= 0.05 and*p_unc_*= 0.016, respectively). Moreover, the peak of decoding accuracies was around the moment of VOE and reduced after the explanation of the tricks (seeFig. 5C).

**Fig. 5.**
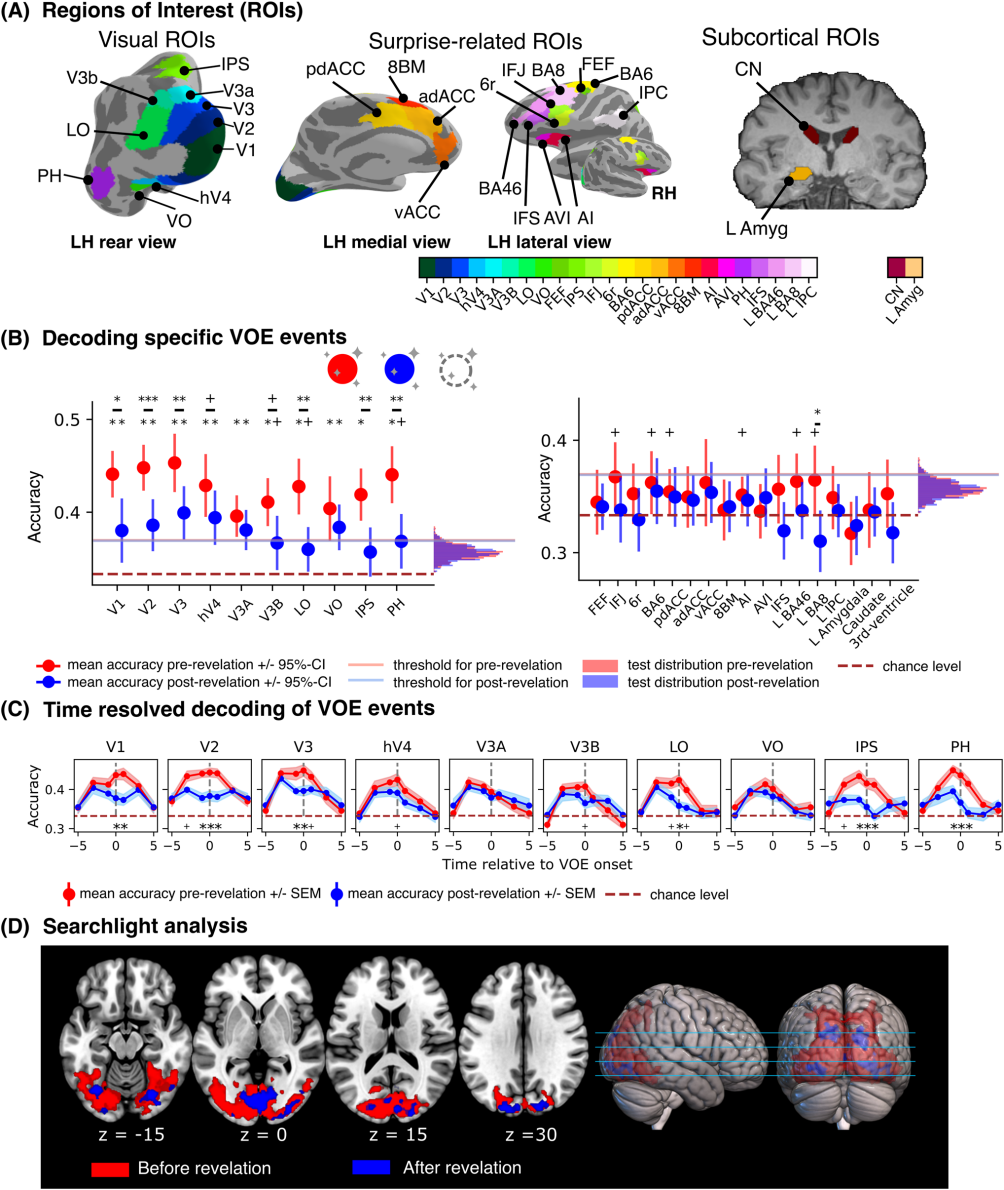
Results of decoding analyses (A), regions of interest (ROIs) used in the experiment. Visual ROIs are shown left, all of which (except for the inferior temporal area PH) were defined using a probabilistic map of visual areas (Wang et al., 2015). Surprise-related ROIs presented on the right panel were defined based on previous results (Danek et al., 2015;Parris et al., 2009) using a multimodal parcellation atlas (Glasser et al., 2016), except for frontal eye field (FEF), which was also defined using the probabilistic atlas fromWang et al. (2015)(see Supplementary Section*Surprise-related region of Interest definition*for more information). The two subcortical ROIs, caudate nucleus, and left amygdala were defined using the Freesurfer automatic parcellation (Fischl et al., 2002). (B), shown are the decoding accuracies for decoding the VOE types over objects in a three-fold cross-decoding approach in our theory-driven ROIs (left: ROIs that significantly decoded the VOE type using pre-revelation data, right: ROIs that did*not*significantly decode the VOE type using pre-revelation data). Decoding was performed with data before (red) and after (blue) revelation. All statistics were corrected for multiple tests by using the max-statistic correction across all ROIs (Nichols & Holmes, 2002). Decoding accuracies pre and post revelation were compared using paired t-tests. ***p < 0.001, **p < 0.01, *p < 0.05, Holm-Bonferroni corrected;^+^p < 0.05, uncorrected. (C), temporal development of decoding accuracies relative to VOE onset. Decoding was performed using beta estimates that modelled moments 5 s before to 5 s after VOE onset in steps of 2 s (-5, -3, -1, +1, +3 +5 s with respect to the VOE onset at 0 s). Decoding accuracies pre-revelation peak around VOE onset and are significantly higher than decoding post-revelation. All statistics were corrected for the number of time steps. *p < 0.05, Holm-Bonferroni corrected;^+^p < 0.05, uncorrected. (D), whole-brain searchlight decoding results. We can significantly decode VOE types in the majority of visual cortex before revelation and less so after revelation (correcting using a permutation-bootstrap hybrid method,Stelzer et al., 2013).

#### 3.5.2 Surprise related ROIs

In contrast, no surprise-related ROI (nor the control ROI) showed significant above-chance decoding accuracies between VOE types using the permutation-max statistic for correction, except for the PH ROI (*p_corr_*< 0.001), an area located in the inferior temporal sulcus (temporo-occipital division), using data before revelation of the tricks. Uncorrected significant above chance decoding (chance level = 33%) was observed in IFJ, AI, pdACC, BA6, BA8, and BA46 using data before revelation. Differences in decoding accuracies before and after revelation were observed only in PH (*p_corr_*= 0.005) (seeFig. 5BandSupplementary Table S20) and in BA8 (corrected for the number of ROIs that could*not*significantly decode the VOE type using pre-revelation data, i.e., 16 ROIs) (seeFig. 5BandSupplementary Table S21).

Therefore, while frontal and parietal areas showed a generic and surprise-dependent involvement in processing VOE in the univariate analyses, they carried no or only weak information (i.e., in BA8) as to what exactly happened. In contrast, information about specific VOE was observed in all posterior sensory areas across the visual hierarchy.

#### 3.5.3 Searchlight analysis

Consistent with the ROI analysis, results of the whole-brain searchlight analysis revealed that only posterior visual areas of the brain could significantly decode the magic type in both conditions (using a permutation-bootstrap hybrid correction method,Stelzer et al., 2013). As shown inFigure 5D, significant decoding was possible in large areas of the visual cortex, including most of the occipital cortex and small parts of the temporal and parietal cortex. Crucially, decoding accuracies before the explanation of the magic tricks were significant in more voxels and larger clusters compared to after explanation. Significant decoding before the explanation of the tricks extends to parts of temporal and parietal cortex, whereas significant decoding after revelation is largely restricted to posterior visual areas. In sum, and in contrast to the univariate results that showed no net modulation as a function of prior knowledge (and surprise) in visual areas, the differences in decoding accuracies using data before and after explanation of the tricks suggest that visual areas are, indeed, sensitive to changes in knowledge and encode specific expectations.

#### 3.5.4 Controlling for time effects in pattern activity

Arguably, suprathreshold decoding of magic effects in posterior sensory areas may reflect general stimulus differences in the moment of magic between the different magic trick types independent of the object used, as appear videos systematically showed red objects, color changing videos blue objects, and vanishing videos no object. However, the observed significant differences in decoding accuracy before and after revelation suggest surprise-dependent modulations of activity patterns, as these differences are present in view of the very same videos. Yet, these differences could reflect time- and/or design-related confounds, such as a general decrease of attention and alertness over time. However, since we did not find any significant changes in univariate comparisons in posterior visual areas and we observed a general increase in parts of the dorsal attention network, we believe that our results are not confounded by time and/or design related factors. Nonetheless, we performed control analyses comparing decoding accuracy of objects present or absent in control videos in the pre versus post revelation phase. These analyses showed no modulation of time in control videos (detailed results can be found in the Supplementary Section*MVPA control analyses*).

## 4 Discussion

In this fMRI study, we used naturalistic video stimuli showing magic tricks and matched control actions to investigate responses to violation of expectations (VOE) of deeply held beliefs about the physical world. We used three distinct magic types (object appearance, object disappearance, and feature-change) that were presented with and without prior knowledge about the underlying deceptive methods (i.e., sleights-of-hand). Each magic type was presented using three distinct objects to allow for object-invariant classification of magic types. We looked for 1) generic prediction error responses to perceived violation of physical principles, 2) specific responses to the different magic types, and 3) effects of the viewers’ prior knowledge on prediction error processing, for both generic and specific responses.

Our results revealed a hierarchy of surprise signals. First, we observed generic effects of world-model VOE (i.e., common to all magic types) in several clusters of the prefrontal and parietal cortex (such as the dorsal and ventral ACC and the posterior parietal cortex) and a correlation of their responses with subjective surprise ratings. Then, differential activity specific to the different types of magic was evoked predominately in posterior visual areas of the occipital and parietal cortex. These specific prediction error signals were evident in the univariate analyses and in decoding of the magic types, both of which were largely confined to posterior areas across the visual hierarchy. Finally, following explanation of the tricks, responses were largely unaffected by participants’ knowledge and only decreased in select parts of the network showing generic effects of VOE (midline areas of the default mode network). While net activity in visual areas was not significantly modulated by the prior knowledge, decoding of VOE type-specific signals was sensitive to changes in the participants knowledge, showing decreased decoding when participants knew the tricks. These results suggest that higher-level predictive information affects even the earliest levels of cortical visual processing (V1–V3).

### 4.1 Generic responses to violation of expectations

Witnessing magic events that violate intuitive physical principles evoked activity in a large network of frontal and parietal (dACC, vACC/mPFC, and posterior parietal cortex) and subcortical (caudate nucleus) areas, with no involvement of lower-level sensory areas. This pattern of activity is consistent with that observed in a recent large meta-analysis of surprising events (Fouragnan et al., 2018), and with previous experiments investigating surprise responses using naturalistic videos, such as magic tricks (Danek et al., 2015), computer-generated animations (Bardi et al., 2017), or learned sequences of movements (Schiffer & Schubotz, 2011). Accordingly, our results add to prior evidence showing the key role of the dACC in processing incongruent information (Alexander & Brown, 2011,^2019^) and of the caudate nucleus in signaling unexpected and rewarding events (Schultz et al., 1997;Wittmann et al., 2008;Zink et al., 2003).

Most importantly, our results suggest that higher-level VOE in view of seemingly impossible events are processed similarly to breaches of lower-level expectations, such as the presence of infrequent stimuli, unlikely events (Kim, 2014), or changes in the stimulus sequences (Downar et al., 2000;Grundei et al., 2023). This suggests the existence of a dedicated network including frontal and parietal areas that correlate with subjective surprise signals, that signal the detection of incongruent information in the human brain (irrespective of sensory modality and abstraction level).

### 4.2 Specific responses to violation of expectations

Complementing the generic frontal and parietal involvement in processing naturalistic violations of physical principles, we further looked into the specific effects of the different types of VOE (appear, change, vanish). Specific responses evoked by the different VOE in net activity and multivariate activation patterns (i.e., allowing for a distinction between trick-types) were observed predominately in posterior sensory areas. In contrast, frontal areas revealed no or only weak specific VOE responses. Since this divergent pattern of net activity was only observable when looking into the individual VOE, it is possible that previous studies failed to report the involvement of sensory areas because of pooling responses to different types of VOE (Danek et al., 2015;Liu et al., 2024;Parris et al., 2009).

Previous results using dynamically occluded stimuli report the neural representation of occluded objects in posterior visual areas (Erlikhman & Caplovitz, 2017;Hulme & Zeki, 2007;Olson et al., 2004) and in neurons of the inferotemporal cortex of macaque monkeys (Puneeth & Arun, 2016) at different levels of complexity (e.g., occluded faces selectively engaged the fusiform face areas,Hulme & Zeki, 2007). The observed differential activity in visual areas using naturalistic stimuli in the present study are likely VOE type-specific surprise signals when violating said representations.

The following reasons support this interpretation: First, net responses were not driven by differences in visual content (because they were evident across distinct objects, and additional control contrasts ruled content-driven responses out). Second, decoding did not work in the absence of VOE when we used similar sensory occurrences using data from the matched control videos. Third, prior knowledge significantly reduced decoding accuracies. And finally, most observed responses occurred in functionally specialized regions of the visual cortex. For example, the unexpected appearance of objects evoked activity in the object-responsive LOC and the perception of unexpected colors evoked activity in the ventral color areas of the fusiform gyrus. Both of these are compatible with prior evidence showing increased activity in the LOC upon perceiving unexpected objects (Richter et al., 2018) and in color areas when viewing unexpected colors (Jiang et al., 2016;Stefanics et al., 2019).

Hence, our results suggest that memory-based expectations related to higher-level principles affect visual processing already at the earliest cortical visual processing areas: they encode information about the presence, absence, and features of objects. This is consistent with recent neuroimaging evidence suggesting that responses in early visual areas can be modulated by high-level visual surprise signals (Richter et al., 2024).

### 4.3 Hierarchical prediction errors in naturalistic perception

The observation of surprise-related information in posterior visual areas is in line with a variety of higher-level memory-based signals that have been reported in visual areas, such as memory color (Bannert & Bartels, 2013), scene context (Muckli et al., 2015), scene segmentation (Grassi et al., 2017,^2018^;Scholte et al., 2008), expected visual stimuli (Ekman et al., 2017), and working memory (Harrison & Tong, 2009). Importantly, these signals have been interpreted as evidence of recurrent predictive signals from higher-level areas, as they encoded information that is not thought to originate from V1 (such as memory color, 3D, or Gestalt and scene information). Consistent with this, further studies located corresponding signals in superficial and/or deeper layers of the cortex using laminar fMRI (Aitken et al., 2020;Lawrence et al., 2018;Muckli et al., 2015) or electrophysiological measurements in monkeys (e.g.,Papale et al., 2022;Self et al., 2013).

Together, the current results fall in line with predictive coding theories and extend them to VOE regarding higher-level world models (Friston, 2005;Lee & Mumford, 2003;Rao & Ballard, 1999). We show a clear dissociation: generic responses to VOE in higher-level frontal and parietal areas and segregated surprise responses in functionally specialized lower-level sensory areas (involved in the processing of the expected information). This reflects the hierarchical structure of our internal world model (Clark, 2013;Hohwy, 2014), with frontal and parietal areas involved in representing more abstract aspects of the world (such as object permanency), while sensory areas represent lower-level inferences about the immediate and detailed features (such as color and shapes). Accordingly, the observed surprise-related responses in lower-level sensory areas can be thought of as the product of a mismatch between top-down predictions (“a red ball”) fed back to lower-level areas to be compared with incoming sensory evidence (“a blue ball”) presumably via feedback connections.

### 4.4 Knowledge-dependent modulations

To further investigate these hierarchical VOE responses, we probed how prior knowledge affected them. We hypothesized that providing the participants with knowledge about the mechanics of the trick for each video would avert VOE: why should we be surprised when viewing a disappearing ball when we know how and that the magician is actually hiding it behind his hands?

Surprisingly, while participants’ subjective surprise ratings were significantly reduced after providing them with the explanation of the tricks, net brain responses were almost indistinguishable: areas involved in the processing of unexpected events, such as the dACC, anterior insula, and caudate nucleus (Fouragnan et al., 2018) were systematically active when observing the magic videos even after participants had rational explanations for the tricks. As neural responses were reminiscent to those signaling surprise, it suggests that repeated viewing of explainable events did not prevent VOE.

This intriguing observation likely reflects that people can be moved by things they know to be unreal, such as fictions (i.e., the “paradox of fiction”, seeRadford & Weston, 1975) or magic illusions (i.e., the “paradox of theatrical magic”, seeGrassi et al., 2024). This is akin to how we still perceive visual illusions, even if we know how they work. For example, when a magician convincingly saws someone in half on stage, the audience is genuinely moved by the illusion (i.e., surprised), but do not attempt to prevent it nor call the police (because they know it is unreal). Our results suggest that the compelling perceptual illusions that magic provides are initially appraised as surprising, even with existing prior-information (cf.,Grassi et al., 2024).

In turn, the only areas whose activity decreased following the explanation of the tricks were two midline core areas of the default mode network (DMN), the ventral ACC/mPFC, and the posterior cingulate cortex. Crucially, our control analysis showed that these modulations could not be explained by the repetition suppression (Grill-Spector et al., 2006;Krekelberg et al., 2006) and condition-independent time effects. The modulation of midline core areas of the DMN by prior knowledge is consistent with recent reports, showing their involvement in processing surprising events in movies (Brandman et al., 2021), jokes (Jääskeläinen et al., 2016) and structured events unfolding in time (Baldassano et al., 2018;Regev et al., 2013;Simony et al., 2016). Based on these findings, it has been suggested that the DMN is not to be understood exclusively as an “intrinsic” network (as originally proposed, cf.,Raichle, 2015), but as a dynamic “sense-making” network involved in the creation of rich models of events by integrating incoming information with prior knowledge as they unfold over time instead (Stawarczyk et al., 2021;Yeshurun et al., 2021).

Here, we show that areas of the “sense-making” network may be sensitive to the rational explanation of magic tricks, whereas all other identified surprise-related regions continued to be sensitive to the VOE (even once the tricks were understood). The decreased involvement of areas of the DMN, together with an increase of activity in frontal and parietal areas of the dorsal attention network (DAN), is consistent with a reduction in prediction error (surprise) signals related to narrative understanding (engaging the DMN) and an increase of top-down attention after explanation of the tricks (engaging the DAN).

### 4.5 Further considerations

Finally, in addition to the violation of intuitive physics and related surprise signals, further cognitive processes should be considered for the interpretation of our results. First, as all our stimuli include a human performer, our results could include neural responses related to action understanding and social cognition. Yet, notably, areas commonly related to social cognition, such as the STS and the TPJ (Deen et al., 2015;Haxby et al., 2000;Lahnakoski et al., 2012;Saxe & Kanwisher, 2003), were not involved in the perception of magic tricks. Instead, these areas related to social cognition showed an increased involvement when perceiving VOE based on unusual actions (i.e.,*Unusual_pre_*>*Magic_pre_*, see also*Control_pre_*>*Magic_pre_*). This is consistent with previous studies revealing an involvement of the STS in the perception of social and psychological VOE, such as irrational actions (Brass et al., 2007;Jastorff et al., 2011;Marsh et al., 2014;Shultz et al., 2011;Vander Wyk et al., 2009), further supporting the notion of functionally-specialized surprise signals.

Moreover, magic tricks, and surprising events in general, capture our attention (Horstmann, 2015) and induce curiosity and information-seeking behaviors (Danek et al., 2014;Lau et al., 2020). Hence, we cannot rule out the involvement of these additional cognitive processes during the perception of seemingly impossible events. However, attentional modulation appears not to drive our effects observed in sensory regions, as they revealed no net BOLD modulation between pre- and post revelation, in contrast to the typically strong net attentional modulation observable in these regions (Jehee et al., 2011;Somers et al., 1999;Tootell et al., 1998). Finally, please note that with concern to surprise-related signal in higher-level regions, differences between pre- and post-revelation activity are unlikely to be related to a set-wise decay in attention, as we see an increase of activity in areas of the dorsal attention network (DAN) post-revelation.

### 4.6 Conclusion

We used a naturalistic paradigm to violate deeply held beliefs of our physical world, involving three types of expectation violations (object appearance, color change, and object disappearance). Our results show a hierarchy of surprise signals: generic responses to unexpected events in frontal and parietal areas, and responses specific to the type of VOE in distinct functionally specialized sensory areas. Our results suggest that world-model VOE are processed similarly to other surprising events in dedicated areas of the prefrontal and parietal cortex and striatum, and that core midline areas of the default-mode network decrease their involvement once rational understanding is established. Most importantly, we show that early and functionally specialized areas of the visual cortex encode memory-based predictions about the presence, absence, and features of objects.

## Supplementary Material

Supplementary Material

## Data Availability

Code (for preprocessing, analysis, and visualisations) and preprocessed data for group analyses can be found athttps://osf.io/kn2af/?view_only=067b698a4567441e93b01518a88860a0. Raw MRI data can be provided upon reasonable request.
