# Supplementary material for: Hierarchical surprise signals in naturalistic violation of expectations
Source: Imaging Neurosci (Camb). 2025 Jan 24;3:imag_a_00459. doi: 10.1162/imag_a_00459 (PMC12319862; doi:10.1162/imag_a_00459)
Supplement: Supplementary Material [file imag_a_00459-supp.pdf]

Supplementary Materials:

Hierarchical surprise signals in naturalistic violation of expectations

Vincent Plikat, Pablo R. Grassi, Julius Frack, Andreas Bartels

Supplementary Methods

Sample size

In our experiment, we had a moderate sample size of 24 participants. This sample size allowed for a balanced design across participants and is similar to comparable VOE studies using video stimuli (using magic tricks: Parris et al., 2008: n = 25, Danek et al., 2015: n = 25; using animations: Bardi et al., 2017: n = 23; Liu et al., 2024: Exp. 1 = 17, Exp. 2 = 32). However, while this sample size is common in neuroimaging (Szucs & Ioannidis, 2020), it still risks missing smaller effects in whole-brain analyses and to capture the true extent of suprathreshold clusters (Geuter et al., 2018). Similarly, our sample size of 24 should be sensitive to medium effect sizes of 0.597 at 80% power for two-tailed paired t-tests and an alpha value of 0.05 in our ROI analyses. To reduce the risk of Type II errors, we hence report both, FWE-corrected results as well as uncorrected results with a cluster threshold of k = 30 in whole-brain analyses.

Behavioral evaluation of stimuli

To ensure the suitability of our stimuli and to select the magic tricks to be used in the fMRI experiment, we performed two behavioral experiments with a total of 18 subjects (nine subjects in each experiment). In the first experiment (P1), participants were asked after each video if a magic trick was shown and to rate from 1 to 5 how surprising the content of the video was (1 = not surprising, 5 = very surprising). Moreover, just before the explanation of each trick, participants were asked if they detected or inferred any of the methods used. In the second experiment (P2), we additionally showed a cue in the center of the screen for 500 ms at the beginning of each video that informed if the video would show a magic trick (M) or not (X). This cue served to ensure that the occurrence of magic versus non-magic in a given trial did not contribute to the surprise rating and it was also used in the fMRI experiment. For both experiments, we expected higher surprise ratings for magic videos compared to control videos and higher surprise ratings before than after the explanations of the tricks. Moreover, we tested whether the unusual videos elicited similar surprise ratings as the magic videos to make both conditions comparable, as intended.

In the first experiment, for all but one of the videos less than a third of the participants correctly explained the underlying method of the trick after four repetitions during the first two runs, i.e. the detection rate was 17 ± 16 %, mean ± SD. One magic trick that was easily detected (50 %) was replaced by another trick for the second validation experiment (detection rate in second experiment was 9 ± 12 %, mean ± SD). As expected, participants easily recognized magic tricks in both experiments (P1: 87 ± 8 %; P2: 93 ± 4 %, mean ± SD) and rated them to be more surprising than the control videos both, before (P1: $t(8)$ = 5.12, p_{unc} < .001, Cohen's d = 1.70; P2: $t(8)$ = 8.50, p_{unc} < .001, Cohen's d = 2.83) and after the explanation of the methods (P1: $t(8)$ = 2.46, p_{unc} = 0.02, Cohen's d = 0.82; P2: $t(8)$ = 2.41, p_{unc} = 0.02, Cohen's d = 0.80). Surprise responses to magic were reduced after knowledge of the tricks,

as expected (P1: $t(8)$ = 2.72, p_{unc} = 0.013, Cohen's d = 0.90; P2: $t(8)$ = 7.50, p_{unc} < 0.001, Cohen's d = 2.50). However, magic tricks were rated consistently more surprising than the unusual videos before revelation of the tricks (P1: $t(8)$ = 4.64, p_{unc} < 0.001, Cohen's d = 1.54; P2: $t(8)$ = 4.67, p_{unc} < 0.001, Cohen's d = 1.56) and in the second experiment also after the revelation of the tricks (P2: $t(8)$ = 2.10, p_{unc} = 0.03, Cohen's d = 0.70).

Region of interest definition

See the main text for rationale of ROI selection. First we defined a set of 16 ROIs based on significant responses to magic videos from previous experiments (Danek et al., 2015; Parris et al., 2009). The authors from Danek et al., (2015) kindly provided us with the corresponding parametric maps from their study which were used to guide the ROI definition. We combined individual labels from a multi-modal parcellation of the human cortex (Glasser et al., 2016), which divides each cortical hemisphere of the brain into 180 parcellations, to define the following 14 frontal and parietal ROIs that showed increased activity when viewing magic tricks. Here we list surprise related ROIs (**bold**) and the labels (*italic*) they consist of: a posterior part of the dorsal anterior cingulate cortex (**pdACC -** *p24pr, a24pr, 33pr* and *p32pr*), an anterior part of the dorsal ACC (**adACC -** *d32, a32pr* and *p24*), ventral ACC (**vACC -** *a24, p32* and *s32*), inferior frontal junction (**IFJ -** *IFJa* and *IFJp*), left inferior frontal sulcus (**IFS -** *IFSa* and *IFSp*), Brodman area 6 (**BA6 -** *6a, 6ma* and *i6-8*), inferior premotor subdivision (**6r -** *6r*), **8BM** - *8BM*, anterior insula (**AI –** *MI* and *AAIC*), anterior ventral insula (**AVI –** *AVI*), inferior temporal gyrus temporo-occipital division (**PH –** *PH*), left **BA 46 -** *a9-46v, p9-46v* and *46*, left **BA 8** - *8C, 8Ad, 8BL* and *8Av* and left inferior parietal cortex (**IPC –** *PF* and *PFop*). The remaining two subcortical ROIs (caudate nucleus and left amygdala) were defined using the Freesurfer automatic parcellation (Fischl et al., 2002).

Second, we used a probabilistic map of visual fields (Wang et al., 2015) to defined ten visual ROIs: primary visual cortex (V1), secondary visual cortex (V2), V3, V3A, V3B, human V4 (hV4), lateral occipital and ventral complex (LO and VO, respectively), intraparietal sulcus (IPS) and frontal eye-fields (FEF). All ROIs were defined in native space.

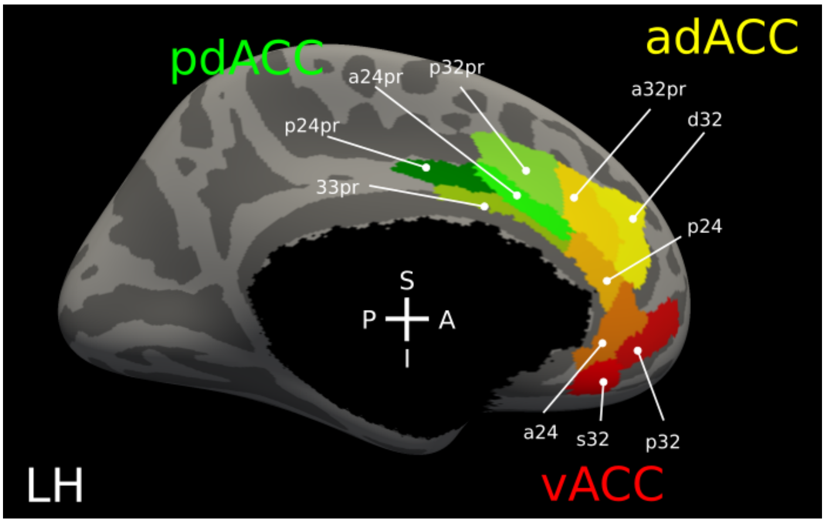

Figure S1. Parcellation of the three anterior cingulate cortex (ACC) ROIs. ACC was divided into a ventral part (vACC), an anterior and posterior dorsal part (adACC and pdACC respectively). All parcellations were taken from multimodal parcellation atlas (Glasser et al., 2016; https://doi.org/10.1038/nature18933).

Eye tracking acquisition and analysis

Data acquisition and preprocessing

Gaze positions were measured using an MR-compatible Eyelink 1000 (SR-Research, Ottawa, Canada) positioned at the rear end of the scanner bore at a 1000 Hz recording rate. Calibration of the eye tracker was performed at the beginning of the experiment and drift correction was performed at the beginning of each run. If necessary, re-calibration was performed at the beginning of a new run.

Eye tracking data (i.e., gaze position, blinks, and saccades) from 23 participants were analyzed. Data from one participant was excluded because of technical problems during data acquisition. Monocular gaze path data (x, y coordinates) were cleaned by removing values 150 ms before and after blinks, linearly interpolating the missing data (with an interpolation limit of 500 ms) and downsampling the data from 1000 Hz to 25 Hz (i.e., the framerate of our videos). Identification of blinks and saccades was performed automatically using the Eyelink online parser with default parameters (saccade detection threshold was 22 degrees/s).

Eye tracking data was used to examine if fMRI responses could be confounded by systematic differences in gaze traces, saccades, or blinks during viewing of the videos. We focused our eye tracking analyses to values within a −1 to +2 s window centered around the event time from each video. We performed two types of tests. First, we used rmANOVAs to test for differences in blink or saccade numbers across conditions. Second, we used correlation analyses to test for differences in gaze traces.

Blink and Saccade analysis

To test for differences in blinks and saccades as a factor of video condition, prior knowledge, and VOE, we compared the mean number of blinks and saccades using two rmANOVAs, for blinks and saccades separately. The first rmANOVA had video (magic and control) and revelation (before and after) condition as factors. The second rmANOVA used magic data only and had the VOE types (appear, color change, and vanish) and revelation (before and after) as factors.

Gaze trace analysis

Finally, to test for differences in gaze traces before and after the revelation of the methods used in the magic tricks, we correlated the x and y positions of each video presentation (eight presentations per video – two per run, two runs pre and post revelation) within a subject, for each video separately (resulting in two 8 × 8 correlation matrices per video – one for x, one for y). Then, the correlation coefficients were transformed using the Fisher-z transformation and averaged for the x and y traces. We pooled all values from comparisons *between* presentations before and after the revelation of the tricks (i.e., the bottom left quadrant of the matrix) and those *within* presentation before and after the revelation (see Figure 2E in the main text for an example) of the tricks within a subject. The pooled correlation coefficients were then compared using paired t-tests.

Supplementary Results

Behavioral results

Table S1: Post-hoc Wilcoxon signed-rank tests on the behavioral surprise ratings of all combinations of video type (magic, control and unusual) and revelation condition (pre- and post-revelation). Significant results are shown in bold. P(corr) show Bonferroni-Holm corrected values.

Contrast	Revelation	Type	A	B	W-val	p(unc)	p(corr)	Cohen's-d
PrePost	-	-	post	pre	0.0	**< .001**	-	-1.364
Type	-	-	Control	Magic	1.0	**< .001**	**< .001**	-3.196
Type	-	-	Control	Surprise	42.0	**0.006**	**0.006**	-0.586
Type	-	-	Magic	Surprise	10.0	**< .001**	**< .001**	1.545
PrePost × Type	post	-	Control	Magic	5.0	**< .001**	**< .001**	-1.554
PrePost × Type	post	-	Control	Surprise	44.0	0.074	0.074	-0.409
PrePost × Type	post	-	Magic	Surprise	26.0	**< .001**	**< .001**	0.812
PrePost × Type	pre	-	Control	Magic	1.0	**< .001**	**< .001**	-4.069
PrePost × Type	pre	-	Control	Surprise	26.0	**0.001**	**0.002**	-0.705
PrePost × Type	pre	-	Magic	Surprise	8.0	**< .001**	**< .001**	2.058
Type × PrePost	-	Control	post	pre	32.0	**0.001**	**0.001**	-0.46
Type × PrePost	-	Magic	post	pre	0.0	**< .001**	**< .001**	-2.166
Type × PrePost	-	Surprise	post	pre	7.0	**< .001**	**< .001**	-0.518

Table S2: Post-hoc paired Wilcoxon signed-rank tests comparing surprise ratings of all magic events before the revelation of the tricks. Significant results are in bold. P(corr) show Bonferroni-Holm corrected values.

Contrast	A	B	W-val	p(unc)	p(corr)	Cohen's-d
Effect	Appear	Change	12.0	**< .001**	**< .001**	-0.835
Effect	Appear	Vanish	35.5	**< .001**	**0.002**	-0.631
Effect	Change	Vanish	98.5	0.235	0.235	0.162

Eye-tracking results

During fMRI measurements we additionally measured saccades and blinks to investigate whether they varied depending on prior knowledge, the video condition and which specific expectation was violated (i.e., unexpected appearance, disappearance or color change).

We performed rmANOVAs with revelation condition (pre and post) and VOE type (appear, color change, vanish) as factors for both, number of blinks and saccades around the moment the VOE occurred (i.e., 1 s before to 2 s after). We did not find any significant difference in the number of blinks between VOE types; however, we observed a significant effect of revelation on the number of blinks ($F(1,22) = 7.67$, $p_{unc} = 0.011$, $\eta^2 = 0.014$, $\varepsilon = 1$). A post-hoc test revealed an increase in mean number of blinks after the revelation of the tricks from 0.82 ± 0.42 to 0.93 ± 0.42, mean ± SD, no significant interaction was found. In contrast, the mean number of saccades showed no systematic difference between pre- and post-revelation runs, but significant differences in the number of saccades of videos showing VOE types ($F(2,44) = 30.73$, $p_{unc} < 0.001$, $\eta^2 = 0.097$, $\varepsilon = 0.996$). These differences probably reflect differences in the content of the videos and are likely neglectable (mean number of saccades

was 6.07 ± 0.9, 5.31 ± 1.00 and 5.69 ± 0.96 mean ± SD for appear, color change, and vanish, respectively). No significant interaction was found.

A further rmANOVA with video (magic and control) and revelation condition (before and after) as factors of the number of blinks around the moment the VOE occurred showed a significant main effect for the revelation condition ($F(1,22)$ = 12.907, p_{unc} = .0016, η^2 = 0.0276) and a significant interaction ($F(1,22)$ = 4.49, p_{unc} = 0.0456, η^2 = 0.002), but not difference between magic and control videos ($F(1,22)$ = 0.003, p_{unc} = 0.95, η^2 = 0.000). Post-hoc t-tests showed that subjects blinked more often post revelation in both, magic and control videos ($t(22)$ = 2.77, p_{unc} = 0.011, Cohen's d = 0.253 and t(22) = 3.639, p_{unc} = 0.001, Cohen's d = 0.398, respectively). The rmANOVA with video and revelation condition as factors of number of saccades around the moment the VOE occurred revealed a significant main effect for the video type ($F(1,22)$ = 7.692, p_{unc} = 0.011, η^2 = 0.014). A post-hoc paired t-test showed that subjects performed more saccades in the control than in the magic videos (t(22) =2.77, p_{unc} = 0.011, Cohen's d=0.243).

Together, these small differences in eye tracking data are unlikely to have affected the imaging results. Since different trick types did primarily involve response differences in specialized visual cortex regions and the number of saccades did not differ pre compared to post revelation it could not cause differences in decoding accuracies pre vs post revelation.

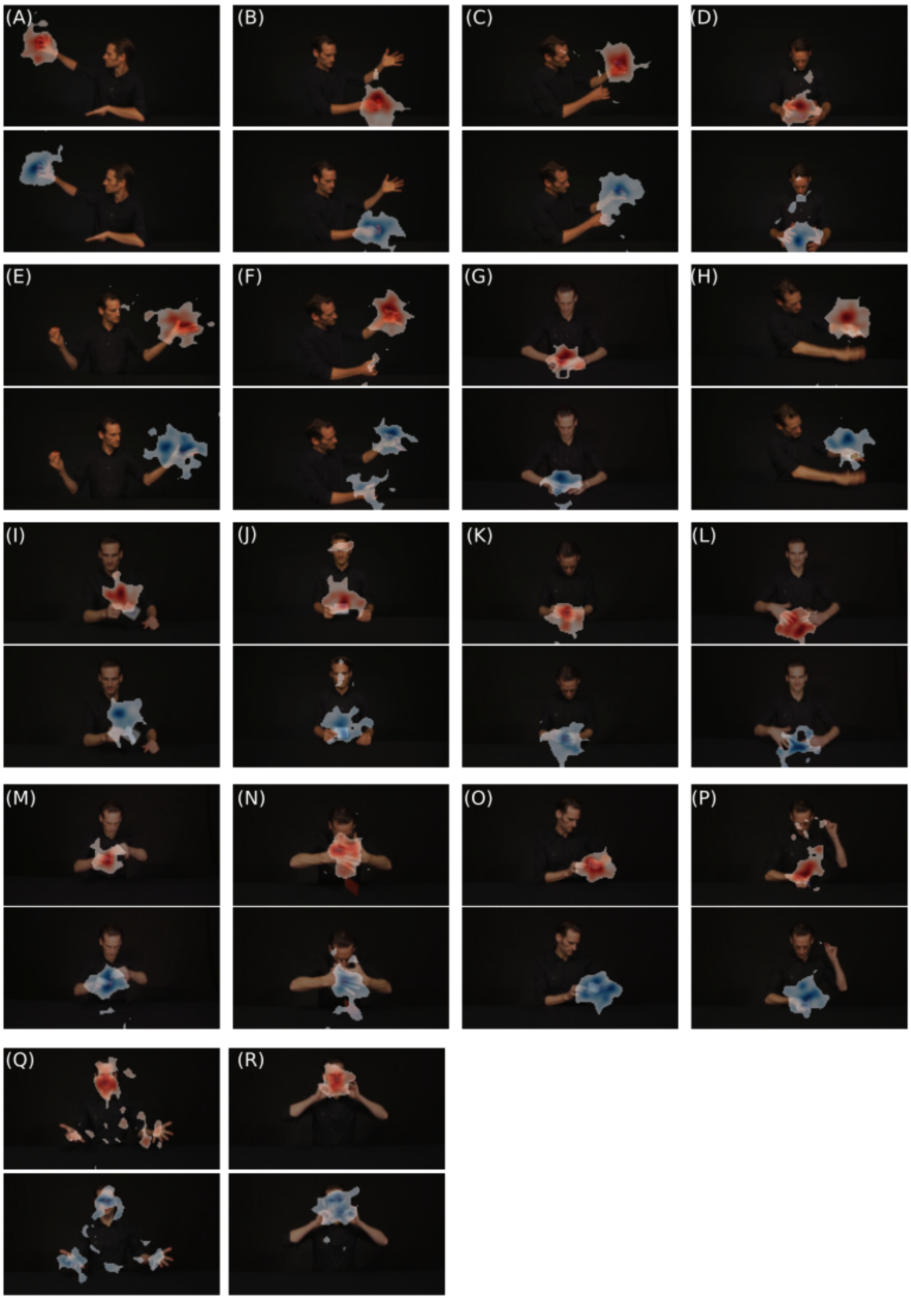

Figure S2. Eye-tracking data with gaze positions before and after the explanation of the tricks. **A-R,** Gaze location around the specific moment in the magic videos (time window -1 s to 2 s around the magic event times) for data before (red) and after (blue). Gaze locations were smoothed with a Gaussian kernel.

Whole-brain fMRI results

Responses to videos showing unusual actions

Analysis of behavioral surprise ratings showed that subjects significantly rated magic videos more surprising than videos showing unusual actions (e.g., the magician eating a playing card) before as well as after revelation. We included the unusual actions with the intention to compare neural responses to seemingly impossible events, that violate physical principles, and responses to surprising actions, that do not violate any physical principles. However, please note that the significant difference in behavioral surprise ratings between magic tricks and unusual actions, as well as the difference in visual content preclude an unconfounded comparison between the two.

As shown in Figure S3, contrasting magic against the unusual actions ($Magic_{pre}$ > $Unusual_{pre}$, Figure S3A) revealed significant frontal (dACC) and parietal (PPC) areas consistent to those observed when contrasting magic against matched control videos. Moreover, the right anterior insula and large portions of the visual cortex showed differential activity. Interestingly, these largely overlaps with the contrast between control videos and unusual actions. (Figure S3B). This could be indicative that the responses observed may not be driven by surprise, but other factors instead (e.g., difference in visual content). A detailed report of these contrasts can be found in Tables S3-6.

Finally, the inverse contrast $Unusual_{pre}$ > $Magic_{pre}$ reveal several clusters of activity (see full list in Table S5), including areas related to social cognition, such as the superior temporal sulcus (STS), the temporo-parietal junction (TPJ) and the ventro-medial prefrontal cortex (vmPFC) (Deen et al., 2015; Hiser & Koenigs, 2018; Saxe & Kanwisher, 2003). This wide-spread activity in areas related to social cognition is likely reflecting social VOE in view of an agent performing unusual and unexpected actions (e.g., showing and eating a playing card), consistent with previous neuroimaging experiments with unexpected or irrational actions (see e.g., Brass et al., 2007; Jastorff et al., 2011; Marsh et al., 2014).

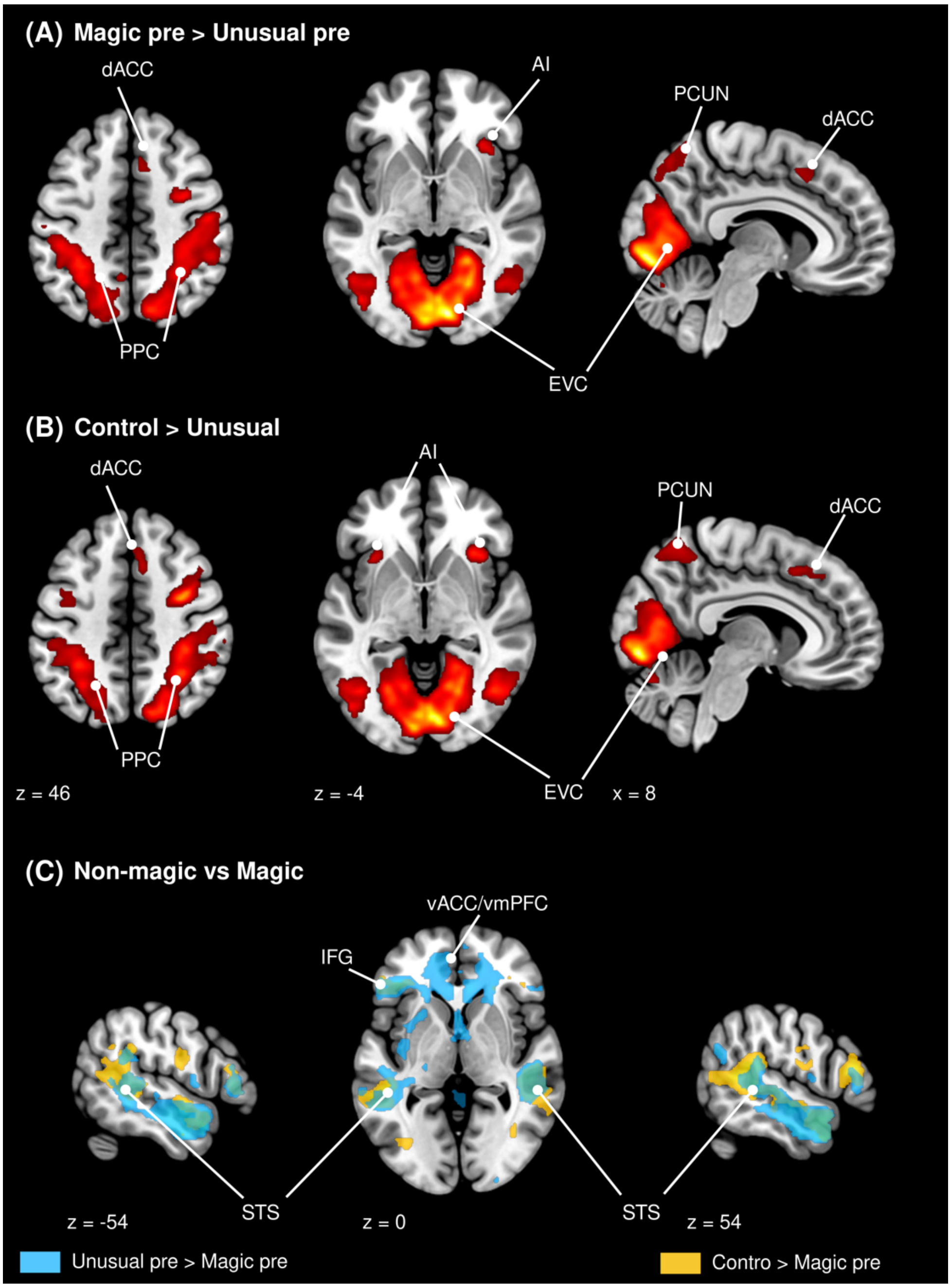

Figure S3. Shown are active areas responding stronger to magic (**A**) and areas responding stronger to control videos (**B**) compared to unusual actions. Both contrasts show largely overlapping areas, predominantly in occipital and parietal areas. **(C)** Shown are areas that respond stronger in the two control conditions ($Control_{pre}$ and $Unusual_{pre}$) compared to magic. Interestingly, both contrasts show involvement of large areas of the superior temporal sulcus. **Abbreviations:** ACC: anterior cingulate cortex; dACC: dorsal ACC; ; PPC: posterior parietal cortex; AI: anterior Insula; EVC: early visual cortex; STS: superior temporal sulcus; IFG: inferior frontal gyrus; vACC: ventral ACC; vmPFC: ventro-medial prefrontal cortex.

Table S3: Results of whole-brain contrast, comparing neural responses between Magic videos and unusual actions before revelation ($Magic_{pre}$ > $Unusual_{pre}$ at p ≤ 0.001 and k = 30, uncorrected). P-values show cluster statistics. Significant permutation-based FWE-corrected results are shown in bold. k = cluster size, *T* = t statistic at peak voxel, x, y, z = peak voxel MNI coordinates [mm]. EVC: early visual cortex; dACC: dorsal ACC; SMA: supplementary motor area.

Brain region	AAL atlas label	p(FWE)	p(unc)	k	T	x	y	z
**EVC**	Lingual_R	**< 0.001**	< 0.001	20263	17.39	8	-84	-4
**V4**	Lingual_R	-	-	-	17.3	22	-64	-8
**EVC**	Calcarine_L	-	-	-	15.96	-6	-88	-2
**R. anterior Insula**	Insula_R	**0.0414**	0.009	150	5.87	30	24	-4
**R. Precentral gyrus**	Precentral_R	0.0854	0.018	95	5.62	50	6	32
**R. dACC/SMA**	Supp_Motor_Area_R	0.1914	0.043	53	4.12	8	16	46

Table S4: Results of whole-brain contrast, comparing neural responses between control videos and unusual actions (*Control* > *Unusual* at p ≤ 0.001 and k = 30, uncorrected). P-values show cluster statistics. Significant permutation-based FWE-corrected results are shown in bold. k = cluster size, *T* = t statistic at peak voxel, x, y, z = peak voxel MNI coordinates [mm]. EVC: early visual cortex, FEF: frontal eye fields, BA: Brodmann area, IFJ: inferior frontal junction, dACC: dorsal ACC.

Brain region	AAL atlas label	p(FWE)	p(unc)	k	T	x	y	z
**EVC**	Lingual_R	**< 0.001**	< 0.001	19268	18.33	6	-82	-6
	Lingual_L	-	-	**-**	16.86	-8	-82	-12
**R. V4**	Lingual_R	**-**	-	**-**	16.02	22	-64	-8
**R. FEF**	Precentral_R	**0.003**	< 0.001	2208	12.4	32	-4	50
**R. BA 6**	Precentral_R	**-**	-	**-**	7.47	50	8	36
**R. IFJ**	Frontal_Inf_Oper_R	**-**	-	**-**	7	38	8	26
**R. anterior Insula**	Insula_R	**0.047**	0.009	169	7.6	32	24	-4
**L. inferior**	Frontal_Inf_Oper_R	**0.008**	0.001	773	6.17	-46	6	28
**premotor**	Precentral_L	**-**	-	**-**	5.26	-42	0	38
**L. FEF**	Precentral_	**-**	-	**-**	4.91	-36	-2	54
**L. anterior Insula**	Insula_L	0.146	0.034	67	5.96	-32	20	-2
**R. dACC**	Cingulum_Mid_R	0.103	0.023	88	5.1	8	16	46
**R. 8BM**	Frontal_Sup_Medial_R	**-**	-	**-**	3.84	4	34	40
**R. inferior frontal cortex**	Frontal_Inf_Orb_R	0.272	0.069	39	4.69	50	40	-10

Table S5: Results of whole-brain contrast, comparing neural responses between unusual actions and magic videos before revelation (*Unusual$_{pre}$* > *Magic$_{pre}$* at p ≤ 0.001 and k = 30, uncorrected). P-values show cluster statistics. Significant permutation-based FWE-corrected results are shown in bold. k = cluster size, *T* = t statistic at peak voxel, x, y, z = peak voxel MNI coordinates [mm]. TPC: Temporal polar cortex; mPFC: medial prefrontal cortex; EVC: early visual cortex; IPC: inferior parietal cortex; TPOJ: Temporo-parieto-occipital junction; SMA: supplementary motor area; BA: Brodmann area; DLPFC: dorsolateral prefrontal cortex; dACC: dorsal ACC.

Brain region	AAL atlas label	p(FWE)	p(unc)	k	T	x	y	z
**R. TPC**	Temporal_Pole_Mid_R	**< 0.001**	< 0.001	31380	12.06	50	8	-30
**L. mPFC**	Frontal_Sup_Medial_L	-	-	-	10.94	-6	48	36
**R. Amygdala**	Hippocampus_R	-	-	-	10.85	22	-10	-12
**R. EVC**	Lingual_R	**0.034**	0.007	193	7.7	22	-96	-12
**R. V4**	Occipital_Inf_R	-	-	-	7.26	34	-90	-12
**R. IPC**	Angular_R	**0.02**	0.003	295	6.31	54	-64	30
	Angular_R	-	-	-	5.22	44	-68	42
**R. TPOJ**	Temporal_Mid_R	-	-	-	4.27	46	-54	20
**L. IPC**	Angular_L	**0.019**	0.003	302	5.81	-46	-66	42
	Angular_L	-	-	-	5.45	-38	-54	26
**R. V2**	Calcarine_R	0.065	0.013	124	5.63	4	-94	14
**R. V3**	Cuneus_R	-	-	-	5.42	10	-94	24
**L. V2**	Occipital_Sup_L	-	-	-	5.31	-12	-98	14
**R. BA 45**	Frontal_Inf_Tri_R	**0.042**	0.009	164	5.29	58	26	14
	Frontal_Inf_Tri_R	-	-	-	5.12	46	32	6
	Frontal_Inf_Tri_R	-	-	-	5.02	56	30	4
**L. V2**	Lingual_L	0.093	0.020	94	5.27	-28	-94	-12
**L. V4**	Lingual_L	-	-	-	5.1	-34	-88	-14
**L. V1**	Occipital_Inf_L	-	-	-	4.13	-22	-100	-6
**L. Precentral gyrus**	Postcentral_L	**0.01**	0.002	508	4.86	-22	-28	64
	no_label	-	-	-	4.82	-22	-30	56
	Paracentral_Lobule_L	-	-	-	4.69	-10	-16	70
**SMA**	Supp_Motor_Area_L	0.163	0.039	62	4.76	-2	16	66
	Supp_Motor_Area_R	-	-	-	3.87	8	14	68

Table S6: Results of whole-brain contrast, comparing neural responses between unusual actions and control videos (*Unusual* > *Control* at p ≤ 0.001 and k = 30, uncorrected). P-values show cluster statistics. Significant permutation-based FWE-corrected results are shown in bold. k = cluster size, *T* = t statistic at peak voxel, x, y, z = peak voxel MNI coordinates [mm]. EVC: early visual cortex; SMA: supplementary motor area; IPC: inferior parietal cortex; IFS: inferior frontal sulcus; BA: Brodmann area.

Brain region	AAL atlas label	p(FWE)	p(unc)	k	T	x	y	z
**R. Amygdala**	no_label	**< 0.001**	< 0. 001	42058	15.7	20	-4	-10
**L. Hippocampus**	Hippocampus_L	-	-	-	13.83	-24	-22	-14
**Thalamus**	no_label	-	-	-	12.73	0	-10	6
**R. Cerebellu**m	Cerebelum_6_R	**0.01**	0.0016	626	7.23	40	-50	-26
	Cerebelum_6_R	-	-	-	6.68	30	-50	-30
	no_label	-	-	-	6.44	26	-38	-32
**R. EVC/V4**	Lingual_R	**0.0312**	0.0063	215	6.75	22	-96	-12
	Occipital_Inf_R	-	-	-	6.74	30	-94	-10
**L. EVC/V4**	Occipital_Inf_L	0.0776	0.0174	108	5.87	-28	-94	-12
	Lingual_L	-	-	-	5.73	-34	-88	-14
**L. V1/V2**	Cuneus_L	**0.0486**	0.0105	155	5.54	4	-94	14
	Occipital_Sup_L	-	-	-	5.4	-12	-98	14
**R. V3**	no_label	-	-	-	5.36	6	-92	22
**R. SMA**	Paracentral_Lobule_R	0.1808	0.043	58	5.53	8	-22	72
	Supp_Motor_Area_R	-	-	-	4.82	8	-16	66
**R. BA 43**	Rolandic_Oper_R	0.2398	0.0614	44	5.37	52	0	10
**R. IPC**	Angular_R	0.1014	0.0226	91	4.75	46	-68	40
	Angular_R	-	-	-	4.74	46	-60	28
**L. IFS**	Frontal_Inf_Tri_L	0.2244	0.0565	47	4.66	-48	28	6

VOE-specific conjunction analyses

To investigate neural responses to specific violations of expectations (VOE) (object appearing, changing color, or vanishing), we performed VOE-specific conjunction analyses, contrasting one specific VOE-type against the two others (e.g., $Magic_{A\_pre} > Magic_{C\_pre} \cap Magic_{A\_pre} > Magic_{V\_pre}$). We observed suprathreshold clusters predominantly in posterior sensory areas (see Table S7).

Table S7: Significant clusters of activity from whole-brain conjunction analyses in which responses to one specific VOE were significantly stronger than in the other two pre revelation ($p \leq 0.001$ and cluster threshold $k = 30$, uncorrected). P-values show cluster statistics. Significant permutation-based FWE-corrected results are shown in bold. k = cluster size, *T* = t statistic at peak voxel, x, y, z = peak voxel MNI coordinates [mm]. Analyses focus on the time point of the magic effect happening. EVC: early visual cortex; IPS: intraparietal sulcus; MT: medial temporal cortex; IT: inferior temporal cortex; SPL: superior parietal lobule; SFG: superior frontal gyrus; IFJ: inferior frontal junction; DLPFC: dorsolateral prefrontal cortex; SMA: supplementary motor area.

Brain region	AAL atlas label	p(FWE)	p(unc)	k	T	x	y	z
**Appear:**								
**R. EVC**	Calcarine_L	**< 0.001**	< 0.001	1390	8.08	4	-90	-4
	Lingual_R	-	-	-	8.04	8	-84	-10
**L. EVC**	Calcarine_L	-	-	-	7.8	-6	-86	-10
**R. V4**	Occipital_Mid_R	**< 0.001**	< 0.001	491	6.49	30	-88	22
**R. IPS**	Occipital_Mid_R	-	-	-	4.53	30	-80	32
	Occipital_Sup_R	-	-	-	4.09	22	-72	44
**L. IPS**	Parietal_Sup_L	**< 0.001**	< 0.001	1450	6.36	-22	-78	44
**L. Occipito-Temporal cortex**	Occipital_Mid_L	-	-	-	6.34	-40	-68	6
**L. IPS**	Occipital_Sup_L	-	-	-	6.23	-24	-82	34
**R. MT**	Temporal_Mid_R	**< 0.001**	< 0.001	409	6.12	46	-66	2
**R. IT**	Temporal_Inf_R	-	-	-	3.7	56	-64	-10
**L. SPL**	Parietal_Sup_L	**< 0.001**	< 0.001	461	5.93	-22	-54	60
	Parietal_Inf_L	-	-	-	4.98	-26	-50	54
**L. Postcentral gyrus**	Postcentral_L	-	-	-	3.74	-28	-38	58
**R. SPL**	Postcentral_R	**0.019**	0.001	178	4.31	24	-48	62
	Postcentral_R	-	-	-	4.04	30	-40	54
**L. SFG**	Precentral_L	**0.038**	0.003	150	4.21	-26	-10	56
	Frontal_Sup_2_L	-	-	-	3.96	-24	-6	66
**Change:**								
**L. EVC**	Occipital_Mid_L	**0.013**	0.001	193	8.12	-30	-96	-6
**R. EVC**	Occipital_Inf_R	**0.002**	<0.001	275	7.57	30	-96	-4
**L. Fusiform Gyrus**	Fusiform_L	0.09	0.007	117	5.2	-30	-50	-16
	Fusiform_L	-	-	-	4.13	-36	-64	-12
**R. IPS**	Angular_R	**0.02**	0.001	176	5.12	32	-54	44
**R. Fusiform Gyrus**	Fusiform_R	0.262	0.023	78	4.9	32	-54	-14
**L. IPS**	Parietal_Inf_L	0.114	0.009	108	4.69	-28	-60	42
**R. IFJ**	Frontal_Inf_Oper_R	0.09	0.007	117	4.04	42	8	28
**R. DLPFC**	Frontal_Inf_Tri_R	0.284	0.025	75	3.91	50	34	18
**R. IFS**	no_label	-	-	-	3.64	36	32	18
**R. IT**	Temporal_Inf_R	0.751	0.105	36	3.72	52	-50	-16
**Vanish:**								
**R. V2**	Cuneus_R	**0.017**	0.001	181	5.7	12	-86	18
**R. V3**	Cuneus_R	-	-	-	3.69	16	-78	26
**L. V2**	Cuneus_L	**0.025**	0.002	166	4.88	-8	-90	18
**L. V3**	Cuneus_L	-	-	-	4.04	-8	-86	30
**L. V2**	Cuneus_L	-	-	-	3.53	-8	-80	24

**R. STS**	Temporal_Mid_R	0.062	0.005	131	4.63	54	-38	6
	Temporal_Mid_R	-	-	-	3.51	46	-36	2
**L. V1-V2**	Calcarine_L	0.384	0.036	64	4.54	-20	-64	12
**R. SMA**	Supp_Motor_Area_R	0.284	0.025	75	4.51	12	10	68
	Supp_Motor_Area_R	-	-	-	3.64	10	14	60
**R. V1**	Calcarine_R	0.678	0.085	41	3.73	18	-76	8
**R. V2**	Lingual_R	-	-	-	3.7	14	-62	0
	Lingual_R	-	-	-	3.65	12	-70	2
**R. V2**	Calcarine_R	0.722	0.096	38	3.66	20	-68	16

VOE-specific responses

Further, we report results of single contrast analyses for each specific VOE-type contrasted with its corresponding control condition (see Figure S4 and Table S8).

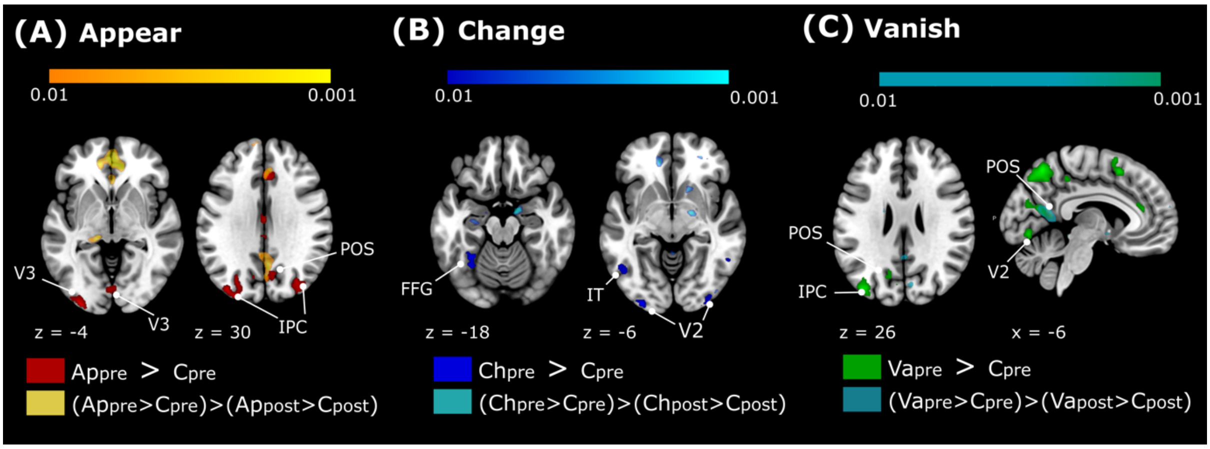

Figure S4. Shown are VOE-specific activations together with their corresponding prior-knowledge dependent contrasts for **(A)** appear, **(B)** color change and **(C)** vanish. Note that none of the posterior sensory areas revealed in VOE-specific contrasts overlap with their corresponding knowledge dependent contrast. Knowledge dependent interaction contrasts are shown as p-value maps, ranging from p_{unc} = 0.01 to 0.001. VOE-specific contrasts show significant clusters with cluster forming threshold of p = 0.001. IPC: inferior parietal cortex; POS: parieto-occipital sulcus; FFG: fusiform gyrus; IT: inferior temporal cortex.

Table S8: Significant clusters of activity from whole-brain contrasts from each VOE type contrasted with its corresponding control condition (thresholded at $p \leq 0.001$ and k = 30, uncorrected). Significant permutation-based FWE-corrected results are shown in bold. IPS: intraparietal sulcus; SPL: superior parietal lobule; FFG: Fusiform gyrus; PCC: posterior cingulate cortex; mPFC: medial prefrontal cortex; SMA: supplementary motor area; DLPFC: dorso-lateral prefrontal cortex; MT: medial temporal lobe; BA: Brodmann area; IT: inferior temporal cortex.

Brain region	AAL atlas label	p(FWE)	p(unc)	k	T	x	y	z
Appear$_{pre}$ > Control$_{pre}$								
**R. inferior parietal cortex**	Parietal_Inf_R	**0.006**	0.0009	599	7.2	46	-38	58
	Postcentral_R				5.99	52	-22	48
**R. anterior IPS**	SupraMarginal_R				5.64	40	-34	46
**SPL**	Precuneus_L	**0.002**	0.0002	2051	7.09	0	-62	50
	Precuneus_L				7.03	-4	-52	46
	Precuneus_L				6.83	-10	-68	46
**L. FFG**	Cerebelum_6_L	**0.013**	0.0019	384	6.81	-22	-56	-16
	Fusiform_L				6.23	-26	-64	-12
**L. Cerebellum**	Cerebelum_6_L				5.54	-30	-46	-24

**L V3**	Occipital_Mid_L	**0.027**	0.4	227	6.32	-32	-94	-4
**L. V4**	Occipital_Mid_L				6.31	-30	-88	2
**L. V1**	Occipital_Inf_L				4.05	-22	-100	-6
**R. inferior parietal cortex**	Occipital_Mid_R	**0.034**	0.0051	195	5.79	32	-76	34
	Occipital_Mid_R				5.52	40	-74	34
	Occipital_Mid_R				5.12	40	-82	24
**R. dorsal ACC**	Cingulum_Ant_R	0.082	0.13	108	5.39	4	28	28
**R. Cerebellum**	Cerebelum_6_R	0.07	0.11	120	5.3	30	-52	-22
**R. FFG**	Fusiform_R				4.32	24	-50	-14
**R. Cerebellum**	Cerebelum_6_R				3.97	36	-50	-30
**R. PCC**	Precuneus_R	0.331	0.0624	38	5.19	8	-44	8
**R. Parieto-occipital sulcus**	Lingual_R				4.82	14	-52	6
**L inferior parietal cortex**	Parietal_Inf_L	**0.048**	0.0078	144	5.07	-44	-34	46
**mPFC**	Frontal_Sup_Medial_L	0.163	0.0263	70	4.95	-2	56	2
	Frontal_Sup_Medial_L				4.52	0	62	8
**R. SMA**	Frontal_Sup_R	0.147	0.0236	75	4.75	30	-8	64
**R. DLPFC**	Frontal_Sup_R				4.71	32	0	64
**L. DLPFC**	Frontal_Mid_L	0.273	0.0485	46	4.72	-28	4	66
**L. PCC**	Calcarine_L	0.135	0.0212	80	4.69	-8	-44	6
	Calcarine_L				4.64	0	-58	10
**L. Parieto-occipital sulcus**	Calcarine_L				4.2	-10	-58	8
**V1**	Lingual_L	0.139	0.0221	78	4.59	0	-80	-2
**L. Parieto-occipital sulcus**	Calcarine_L	0.339	0.0646	37	4.45	-14	-64	20
	Cuneus_l				4.13	-10	-70	28
**L. dorsal ACC**	Supp_Motor_Area_L	0.174	0.0282	67	4.4	-4	16	46
**L. SPL**	Parietal_Inf_L	0.288	0.0527	43	4.09	-38	-48	54
$Change_{pre}$ > $Control_{pre}$								
**L. V2**	Occipital_Inf_L	0.235	0.31	56	5.55	-30	-94	-6
**L. V3**	Lingual_L				3.98	-24	-94	-12
**R. MT**	Occipital_Inf_R	0.134	0.0158	83	5.37	50	-64	-12
	Temporal_Inf_R				4.41	54	-54	-10
**R. inferior parietal cortex**	Postcentral_R	**0.02**	0.2	245	5.22	52	-18	42
**R. BA 1**	Postcentral_R				4.91	54	-18	52
**R. BA 2**	Postcentral_R				4.71	42	-30	44
**R. DLPFC**	Frontal_Mid_R	0.184	0.0229	67	5.05	30	8	58
**L. IT**	Temporal_Inf_L	0.14	0.0165	81	4.94	-46	-60	-8
	Occipital_Inf_L				3.76	-52	-70	-10

**L. FFG**	Fusiform_L	0.201	0.0255	63	4.89	-30	-50	-18
	Cerebelum_6_L				4.65	-28	-58	-16
**R. medial intraparietal cortex**	Agular_R	0.3	0.0426	45	4.87	26	-64	48
**R. V2**	Occipital_Inf_R	0.124	0.0144	88	4.74	32	-92	-2
	Occipital_Inf_R				4.23	26	-98	-4
**R. SPL**	Precuneus_R	0.395	0.0621	35	4.58	8	-68	52
**L. dorsal ACC**	Cingulum_Ant_L	0.3	0.0426	45	4.29	-2	30	16
	Cingulum_Ant_R				4.09	6	34	24
	Cingulum_Ant_L				3.62	-10	34	20
$Vanish_{pre}$ > $Control_{pre}$								
**L. inferior parietal cortex**	Occipital_Mid_L	**0.032**	0.0037	207	6.63	-38	-80	28
	Occipital_Mid_L				5.19	-34	-74	32
**L. SPL**	Precuneus_L	**0.011**	0.0013	389	6.57	-6	-62	54
	Precuneus_R				4.46	4	-70	52
**L. Parieto-occipital sulcus**	Calcarine_L	**0.016**	0.0018	331	6.05	-12	-60	18
**L. V2**	Cuneus_L				4.08	-6	-76	20
**L. Parieto-occipital sulcus**	Cuneus_L				3.75	-14	-70	24
**L. SMA**	Frontal_Sup_L	**0.008**	0.0008	464	5.61	-22	12	56
**L. DLPFC**	Frontal_Mid_L				5.09	-22	28	38
**L. premotor cortex**	Frontal_Sup_L				4.97	-24	0	50
**L. SMA**	Supp_Motor_Area_L	0.103	0.13	107	5.42	-4	12	54
	Supp_Motor_Area_L				4.66	-6	10	64
**L. V2**	Lingual_L	0.242	0.0362	54	5.38	-6	-76	-8
**R. DLPFC**	Frontal_Mid_R	0.436	0.0804	30	5.09	28	28	42
**L. DLPFC**	Frontal_Mid_L	0.272	0.0424	48	4.85	-32	54	20
**L. dorsal ACC**	Cingulum_Ant_L	0.223	0.0324	59	4.69	-4	36	16
	Cingulum_Ant_L				3.81	-2	28	20

VOE-specific control analyses

Our VOE-specific analyses may be confounded as the analyses compare different visual inputs, i.e., either a red object, a blue object, or no object at all. To control for this possible stimulus-driven confound we compared responses between magic and control videos showing similar visual contents at specific time points. We contrasted responses to magic appearances with the control videos for vanishing tricks (as both videos show a "red object" at the specific moments), and magic vanishes with the control videos for tricks showing something appear (as both videos show no objects at the specific moments). No similar match was possible for the color-changing videos. Responses similar to that of the original contrasts, which used control videos showing similar actions, would be indicative that the observed differential activity reflects specific surprise responses and not differences in the visual content of the videos.

Results were similar to the corresponding VOE-specific analysis (see Figure S5). Please note that no such control analyses could be performed for the color-changing trick.

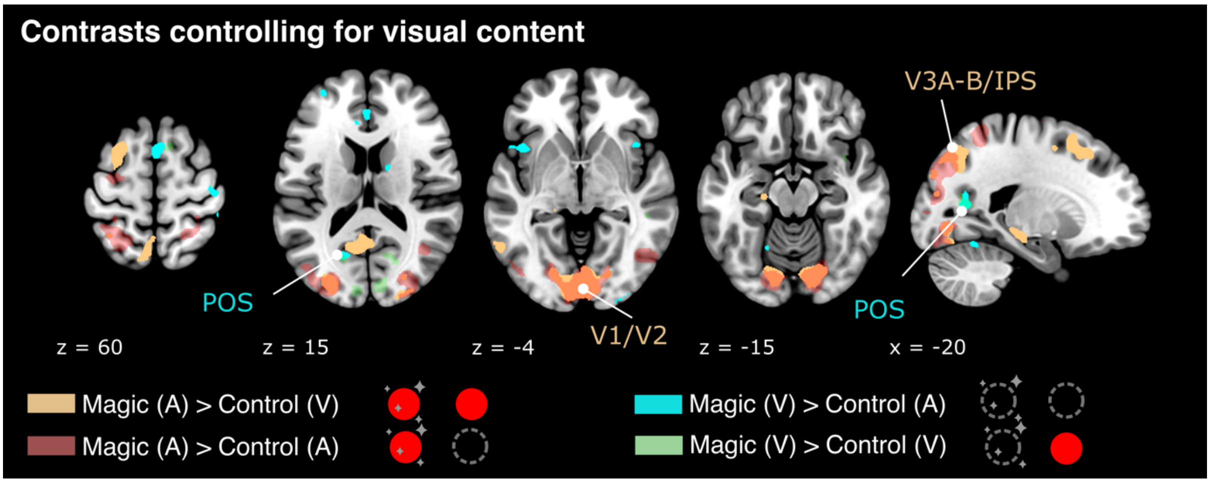

Figure S5. Shown are results of our VOE-specific control analysis, contrasting responses to VOEs resulting in a red object in the scene ($Magic_{app}$) with control videos showing a red object in the scene ($Control_{van}$) as well as contrasting VOEs resulting in no object shown ($Magic_{van}$) with control videos showing no object ($Control_{app}$). Both results are similar to the contrasts of VOEs with their corresponding control videos.

Prior Knowledge dependent control analysis

We investigated how neural responses to seemingly impossible events in the form of magic tricks change with prior knowledge about the working of said magic tricks. We did so, by comparing whole-brain contrasts of the interaction between the prior knowledge condition and the video type condition *(*$Magic_{pre}$ > $Control_{pre}$*)* > *(*$Magic_{post}$ > $Control_{post}$*)*. This contrast controls for possible time confounds, since it weights one condition positive (Magic condition) and one condition negative (Control condition) before revelation and similarly it weights one condition positive (Control condition) and one condition negative (Magic condition) after revelation. However, it might be possible that there is an interaction in the way that with time (not with knowledge) the neural response to magic decreases faster than the one to control actions. Moreover, our knowledge-dependent analysis could be confounded by the repetition suppression effect (Grill-Spector et al., 2006; Krekelberg et al., 2006), as participants were repeatedly presented to the same video stimuli within a set.

To control for condition-independent time effects and the repetition suppression effect, we extracted beta estimates corresponding to all magic and matched control presentations from the suprathreshold clusters, averaged them over subjects and calculated the same contrast within each run separately. We ran a mixed-effects model on the contrast values with three predictors: a pre-post predictor (i.e. 1, 1, -1, -1 for each set), a constant decay predictor over all 12 fMRI runs (i.e. 5.5 to -5.5 in 12 steps) and a constant decay predictor within one set (i.e., 1.5 to –1.5 in four steps for each set). Significant neural differences driven by knowledge should show a significant pre-post predictor. In turn, any time-dependent variance should be accounted for by the experiment-wise and set-wise decay predictors. Results are shown in Figure S6 and listed in table S9. All suprathreshold clusters had significant pre-post predictor.

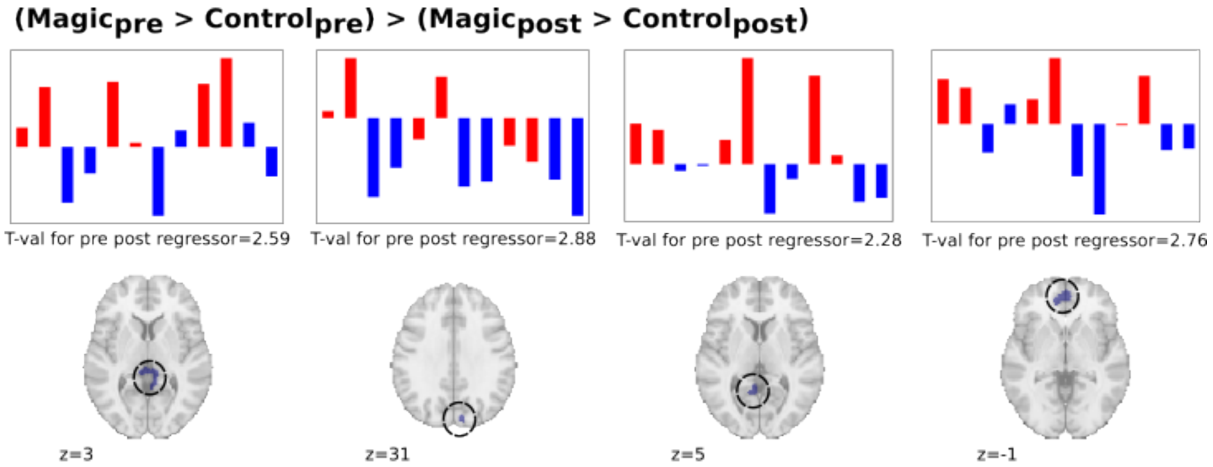

Figure S6. Shown the differences between beta estimates of the Magic and corresponding control condition for each run. Red bars are runs pre revelation, blue bars are post revelation runs. The results show that beta estimates are not decreasing constantly over time but drop after learning the method behind the magic tricks shown instead.

Table S9: Results of a mixed effects model from the difference between magic-control in each experimental run averaged over subjects and a regressor that tests for the influence of prior knowledge as well as multiple regressors controlling for knowledge independent time confounds. The table shows t-values with corresponding p-values for each regressor. The knowledge dependent regressor (pre post) was significant in all clusters. Pre post: knowledge dependent regressor, set decline: temporal decline within one set of objects, runs: temporal decline over the course of the whole experiment.

AAL Anat. area	Pre post T	Pre post p	Pre post p_{corr}	Set decline T	Set decline p	Runs T	Runs p
**R Lingual**	2.59	**.009**	**.019**	-0.39	.700	-1.854	.064
**R Cuneus**	2.883	**.003**	**.016**	-1.306	.191	2.116	**.034**
**L Calcarine**	2.283	**.022**	**.022**	-0.454	.650	0.48	.631
**L Cingulate Ant**	2.759	**.005**	**.017**	-1.082	.280	0.946	.344

Neural activity following explanation of the tricks

Responses to magic after revealing the method behind the magic tricks ($Magic_{post}$ > $Control_{post}$) were similar to those before the revelation of the tricks. A detailed list is shown in Table S10.

Table S10: Significant clusters of activity from the whole-brain contrast comparing responses between magic videos and matched controls after revealing the method behind the magic tricks ($Magic_{post}$ > $Control_{post}$, thresholded at $p \leq 0.001$ and k = 30, uncorrected). P-values show cluster statistics. Significant permutation-based FWE-corrected results are shown in bold. k = cluster size, *T* = t statistic at peak voxel, x, y, z = peak voxel MNI coordinates [mm]. PCC: posterior cingulate cortex; dACC: dorsal anterior cingulate cortex.

Brain region	AAL atlas label	p(FWE)	p(unc)	k	T	x	y	z
**L. Posterior parietal cortex**	Precuneus_L	0.0016	0.0002	4484	8.35	-10	-72	42
	Parietal_Inf_L				7.94	-42	-36	42
	Precuneus_L				7.72	-6	-72	52
**PCC**	Cingulum_Post_L	0.013	0.0023	468	7.26	-6	-42	22
	no_label				6.38	-4	-28	26
	no_label				4.95	4	-18	30
**R. Superior frontal gyrus**	Frontal_Sup_R	0.0188	0.0035	323	7.1	28	2	58
	Frontal_Sup_R				3.73	30	-4	64
**L. Superior frontal gyrus**	Frontal_Mid_L	0.0084	0.0015	683	6.51	-28	8	56
	Precentral_L				5.13	-28	-4	48
**dACC**	Supp_Motor_Area_R	0.0142	0.0027	425	5.94	6	14	50
	Supp_Motor_Area_R				5.81	-6	10	52
	Cingulum_Mid_R				4.63	-6	26	36
**R. Postcentral gyrus**	Postcentral_R	0.0068	0.0012	835	5.52	52	-26	56
	Postcentral_R				5.47	46	-26	42
	Postcentral_R				5.35	54	-22	42
**R. lateral occipital**	Occipital_Mid_R	0.0416	0.0077	186	5.37	40	-76	28
	Occipital_Mid_R				4.25	38	-84	22
**L. anterior middle frontal gyrus**	Frontal_Mid_L	0.0618	0.0117	137	5.31	-34	58	10
	Frontal_Sup_L				4.43	-28	58	2
**R. Cerebellum**	Cerebelum_6_R	0.225	0.0515	51	5.23	28	-64	-26
**L. inferior temporal**	Temporal_Inf_L	0.2496	0.059	46	5.18	-56	-60	-4
**L. Precentral gyrus**	Precentral_L	0.2496	0.059	46	4.6	-50	6	36
	Precentral_L				3.92	-44	2	26
**R. dACC**	Cingulum_Ant_R	0.3406	0.0893	33	4.57	8	24	30

Whole-brain fMRI results for complete videos

Our main analyses of the whole-brain fMRI data were based on responses to the specific moment in each video where the violation of expectation occurred (and their counterpart in control videos). We further analyzed data over the whole video presentation. Results are remarkably similar to those using discrete event times (see Tables S11-13 and Figure S7). The conjunction analysis of all three magic events did not result in any significant cluster (at threshold $p_{unc} < 0.001$ and k = 30). These results indicate that our main analyses are unlikely to be influenced by the selection of the event times.

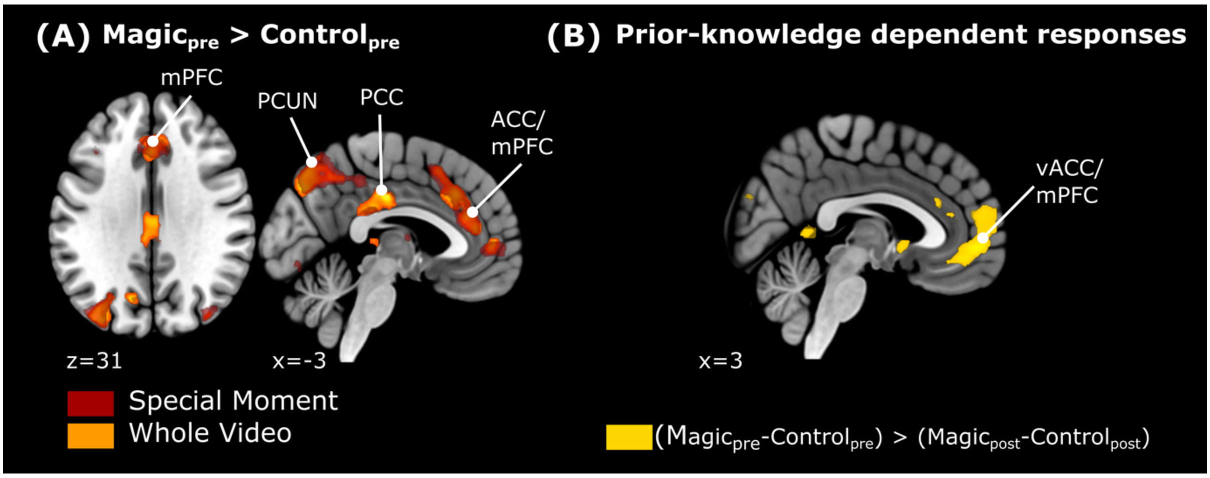

Figure S7. Shown are univariate contrasts resulting from analyses that take a specific moment in which the VOE occurred in consideration and from analyses that take the whole time of video presentation in consideration. (**A**) Magic > Control before. **(B)** Prior knowledge dependent responses.

Table S11: Results of whole-brain contrast, comparing neural responses to magic videos and control videos ($Magic_{pre}$ > $Control_{pre}$) over the whole duration of video presentation (thresholded at $p_{unc} < 0.001$ and k = 30, uncorrected). P-values show permutation-based cluster statistics. K = cluster size, T = t statistic at peak voxel, x, y, z = peak voxel MNI coordinates [mm]. Results mostly reflect the results from analysis only using data from the specific time point. PCC: posterior cingulate cortex, PPC: posterior parietal cortex, dACC: dorsal ACC, aPFC: anterior prefrontal cortex, SFG: superior frontal gyrus, vACC: ventral ACC, aMFG: anterior middle frontal gyrus.

Brain region	Anatomical area	p(FWE)	p(unc)	k	T	x	y	z
**PCC**	Cingulate_Mid_L	**0.0212**	0.0034	325	7.1	-2	-20	36
	no_label	-	-	-	4.73	-4	-30	28
	no_label	-	-	-	4.7	6	-32	26
**Posterior parietal cortex**	Occipital_Mid_L	**0.0014**	0.0001	1728	6.75	-32	-82	34
	Precuneus_L	-	-	-	6.32	-10	-72	52
	Precuneus_R	-	-	-	6.12	4	-70	52
**dACC**	Cingulate_Ant_L	**0.006**	0.0007	862	6.37	-6	34	24
	Cingulate_Ant_R	-	-	-	5.75	6	34	24
	Cingulate_Mid_L	-	-	-	5.56	0	24	34
**L. aPFC**	Frontal_Sup_2_L	0.1134	0.02	103	5.55	-24	58	6
	Frontal_Sup_2_L	-	-	-	3.89	-32	56	-2
**L. SFG**	Frontal_Sup_2_L	**0.0368**	0.0064	217	5.19	-20	12	56
	Frontal_Sup_2_L	-	-	-	4.5	-24	-4	52
	Frontal_Mid_2_L	-	-	-	4.44	-28	8	60
**L. anterior intraparietal**	Parietal_Inf_L	0.0922	0.0161	117	4.84	-38	-40	46
	Parietal_Inf_L	-	-	-	3.64	-32	-44	42
**vACC**	Cingulate_Ant_L	0.1486	0.0271	81	4.84	-2	48	4
**R. Postcentral gyrus**	Precentral_R	0.0666	0.0115	144	4.71	50	-16	46
	Precentral_R	-	-	-	4.08	48	-14	56
**R. Supramarginal**	Postcentral_R	-	-	-	4.06	42	-28	40

**gyrus**								
**R. PCC**	no_label	0.2706	0.0542	48	4.53	10	-36	2
**no label**	Lingual_R	-	-	-	4.4	8	-36	-6
**L. aMFG**	Frontal_Mid_2_L	0.2044	0.0386	62	4.42	-30	50	14
**L. Cerebellum**	Cerebelum_6_L	0.3908	0.0871	33	4.32	-26	-54	-20
**L. Fusiform gyrus**	Fusiform_L	-	-	-	4.04	-24	-62	-14

Table S12: Results of contrasts comparing neural responses to VOEs before and after explanation of the tricks, $(Magic_{pre} > Control_{pre}) > (Magic_{post} > Control_{post})$, over the whole duration of video presentation (thresholded at $p_{unc} < 0.001$ and k = 30, uncorrected). P-values show permutation-based cluster statistics. K = cluster size, T = t statistic at peak voxel, x, y, z = peak voxel MNI coordinates [mm]. Results mostly reflect the results from analysis only using data from the specific time point. vACC: ventral ACC, mPFC: medial prefrontal cortex. PCC: posterior cingulate cortex. dACC: dorsal ACC, BA: Brodmann area

Brain region	Anatomical area	p(FWE)	p(unc)	k	T	x	y	z
**vACC**	Frontal_Med_Orb_R	**0.002**	0.0003	1123	6.03	4	50	-4
**mPFC**	Frontal_Sup_Medial_R	-	-	-	5.26	6	60	6
**vACC**	Frontal_Med_Orb_L	-	-	-	5.25	-12	42	-6
**L. PCC**	Precuneus_L	0.1858	0.0292	68	5.87	-8	-54	10
	Calcarine_L	-	-	-	4.11	-6	-62	12
**R. PCC**	Precuneus_R	0.0832	0.0114	120	5.78	6	-48	6
	Lingual_R	-	-	-	3.89	16	-48	4
**L. Hippocampus**	Hippocampus_L	0.106	0.0149	102	5.66	-28	-16	-14
**L. Parahippocampus**	Fusiform_L	0.1748	0.0272	71	5.36	-24	-36	-18
**L. Hippocampus**	Hippocampus_L	-	-	-	4.77	-20	-30	-6
**R. Parahippocampus**	ParaHippocampal_R	0.3678	0.0743	35	5.03	26	-36	-12
**R. Amygdala**	Hippocampus_R	0.1722	0.0266	72	4.93	18	-6	-16
**no label**	no_label	-	-	-	4.66	8	-2	-10
**L. Nucleus Basalis**	no_label	0.212	0.0346	61	4.67	-4	2	-8
**R. Caudate Nucleus**	no_label	-	-	-	4.51	4	8	-2
**R. V3**	Cuneus_R	0.096	0.0132	110	4.55	8	-86	30
**R. V2**	Cuneus_R	-	-	-	4.22	10	-76	26
**R. V3**	Cuneus_R	-	-	-	3.77	18	-80	32

Table S13: Significant clusters of activity from whole-brain conjunction analyses in which responses to one magic event were significantly stronger than in the other two magic events pre revelation ($p \leq 0.001$ and cluster threshold $k = 30$, uncorrected). P-values show cluster statistics. k = cluster size, T = t statistic at peak voxel, x, y, z = peak voxel MNI coordinates [mm]. Analyses take the whole time of the magic videos into account. EVC: early visual cortex. PPC: posterior parietal cortex; FFG: fusiform gyrus; IT: inferior temporal; IPS: intraparietal sulcus; SMA: supplementary motor area.

Brain region	Anatomical area	p(FWE)	p(unc)	k	T	x	y	z
**Appear conjunction**								
**EVC**	Lingual_R	**< 0.001**	< 0.001	2417	8.22	8	-78	0
	Lingual_L	-	-	-	7.72	-8	-80	-8
	Calcarine_R	-	-	-	6.27	6	-90	0
**L. V4**	Occipital_Mid_L	0.209	0.02	94	4.51	-22	-94	20
**R. PPC**	Parietal_Sup_R	0.762	0.122	37	3.76	16	-76	54
	Precuneus_R	-	-	-	3.72	6	-66	60
**Change conjunction**								
**L. V3/V4**	Occipital_Mid_L	**0.001**	< 0.001	349	7.59	-32	-94	-4
**R. V3/V4**	Occipital_Inf_R	**< 0.001**	< 0.001	392	7.14	34	-92	-4
**L. FFG**	Fusiform_L	**0.001**	< 0.001	327	5.81	-30	-52	-16
	Fusiform_L	**-**	-	-	4.4	-36	-64	-12
**L. IT**	Temporal_Inf_L	**-**	-	-	4.2	-46	-64	-10
**R. FFG**	Fusiform_R	**0.044**	0.004	159	4.58	30	-52	-14
**R. IT**	Temporal_Inf_R	-	-	-	3.82	42	-66	-10
**R. FFG**	Fusiform_R	-	-	-	3.61	32	-60	-12
**R. IT**	Temporal_Inf_R	0.657	0.091	45	4.05	48	-50	-18
**Vanish conjunction**								
**R. SMA**	Supp_Motor_Area_R	0.67	0.095	44	4.47	12	8	68
**L. Supramarginal gyrus**	Postcentral_L	0.079	0.007	134	4.31	-56	-24	30
	SupraMarginal_L	-	-	-	3.81	-64	-26	24
	Postcentral_L	-	-	-	3.56	-58	-18	22

Univariate ROI results

For the univariate ROI analysis we performed the same comparisons as in the whole brain contrast analyses using beta estimates from all ROIs for the magic and control condition and for each magic event (appearances, color changes and vanishes). Estimates were averaged across runs, separately for data before and after revelation of the tricks. First, values from magic and control estimates were used to look for areas generally involved in VOE using paired tests (paired t-tests or Wilcoxon signed-rank test). Then, the difference between VOE-specific contrasts values were used in a 2 x 3 (*revelation x VOE type*) rmANOVA to test for VOE-specific responses and for possible modulations by prior knowledge. Results are shown in Figure S8A and listed in Tables S14-16.

In line with the whole-brain analyses, we observed VOE responses in frontoparietal (IPS, ACC and BA46) and subcortical areas (caudate nucleus). Only the ACC, insula and amygdala ROIs were modulated by prior knowledge (the latter two were not observed in the whole-brain analysis). No visual ROI showed a generic response to VOE (see Table S14) nor modulation by prior knowledge (except for V3B, see Table S15). Yet, all visual ROIs (except for FEF) showed a significant main effect for the VOE type in our rmANOVA with VOE-types and revelation condition as factors (see Table S16). Importantly, none of the visual ROIs showed a significant interaction between VOE-type and revelation condition, in line with our whole brain analyses. This suggests that net activity in posterior sensory areas are sensitive to specific types of VOE, but relatively unaffected by prior knowledge.

As some of the ROI results revealed strong resemblances in their response patterns, we performed a clustering analysis using the correlation distance between them to quantity their similarities and group them. For the cluster analysis we averaged the response in a ROI over subjects for each magic effect minus its corresponding control condition, before and after revelation, separately, resulting in six values per ROI and used these values to calculate the correlation distances ($1-r$) for all ROI pairs. The resulting dendrogram reveals two groups of visual areas, all early visual areas (V1, V2, V3 and V3A) and mid- to high-level visual areas (LO, hV4, VO, V3B, IPS, including PH) (see green and brown clusters in Figure S8B, respectively). In turn, surprise-related ROIs build several subgroups, such as the aACC and the mACC (red cluster) and the BA8, 8BM and Caudate Nucleus (orange cluster).

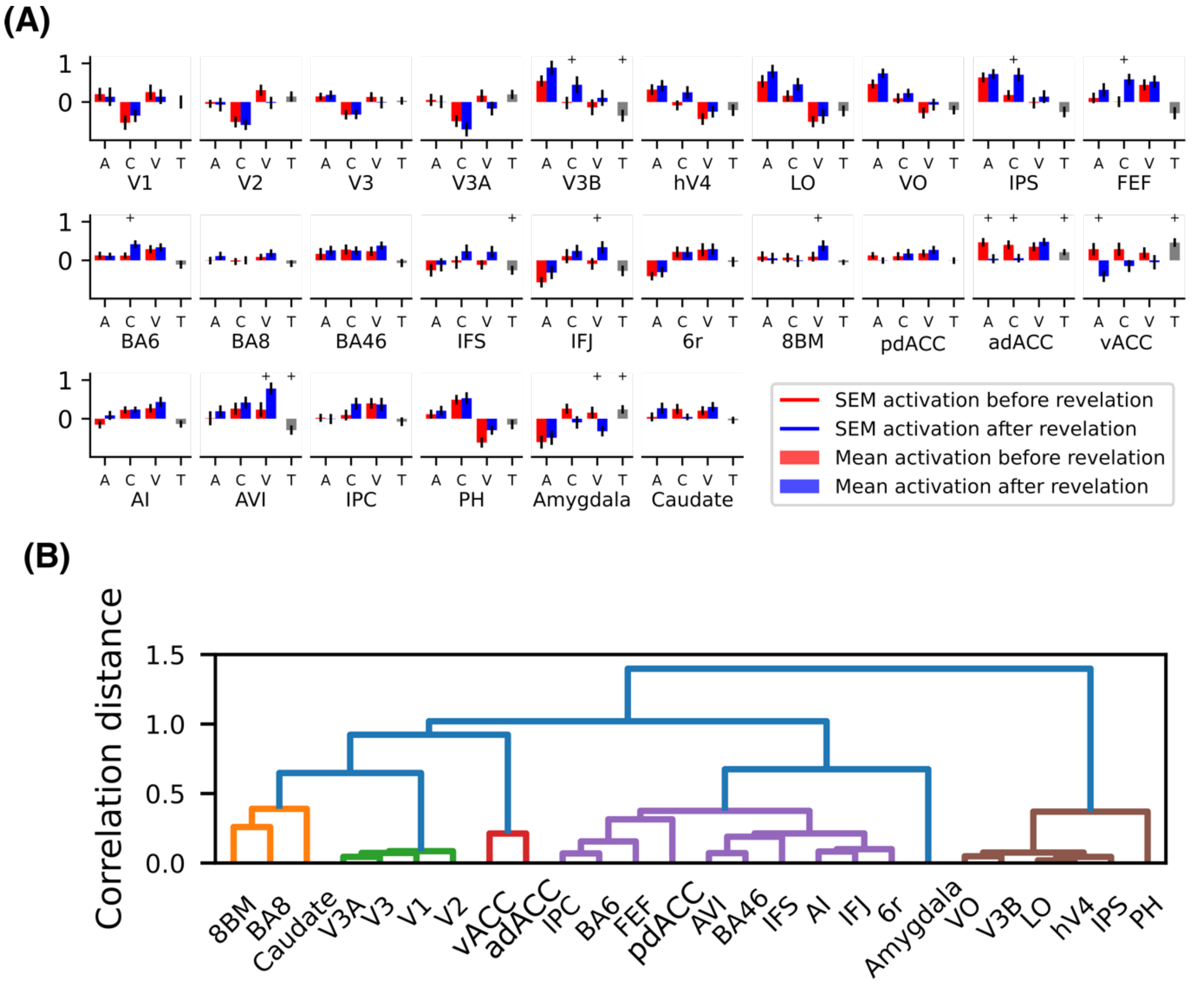

Figure S8. (A), shown are estimates for each VOE type minus the corresponding control condition before and after revelation (e.g., $Magic_{A\_pre}$ – $Control_{A\_pre}$) for each ROI. Red and blue bars show activation before and after revelation respectively for each VOE type (appearances, A; color changes, C; vanishes, V); gray bars show the total pooled prior knowledge modulation (T), i.e., *($Magic_{pre}$ > $Control_{pre}$) > ($Magic_{post}$ > $Control_{post}$)*. **(B)**, Correlation distance dendrogram visualizing the clustering of ROIs based on activation during the perception of different VOE types minus their corresponding controls.

Table S14: Comparing activation during the perception of magic tricks and control videos pre revelation in Regions of Interest. Data was tested for normality, in case normality was violated, we performed a Wilcoxon signed-rank test, otherwise we performed an undirected t-test for dependent measurements. P-values were Bonferroni-Holm corrected.

ROI	test	statistic	p-val	p-corrected	effect-size
**V1**	t-test	-0.353	0.727	1	0.05
**V2**	t-test	-1.146	0.264	1	0.141
**V3**	t-test	-0.249	0.805	1	0.038
**hV4**	t-test	-0.828	0.416	1	0.13
**V3A**	t-test	-0.843	0.408	1	0.12
**V3B**	t-test	1.08	0.291	1	0.148
**LO**	t-test	0.483	0.634	1	0.052
**VO**	t-test	1.189	0.247	1	0.145
**IPS**	t-test	2.746	**0.012**	0.299	0.53
**FEF**	t-test	1.693	0.104	1	0.314
**IFJ**	t-test	-1.646	0.113	1	0.342
**6r**	t-test	0.324	0.749	1	0.069
**BA6**	t-test	2.417	**0.024**	0.527	0.649
**pdACC**	t-test	2.182	**0.04**	0.831	0.435
**adACC**	t-test	5.32	**< 0.001**	**0.001**	0.917
**vACC**	Wilcoxon	69	**0.019**	0.447	0.517
**8BM**	t-test	0.963	0.345	1	0.189
**AI**	Wilcoxon	98	0.143	1	0.233
**AVI**	t-test	1.275	0.215	1	0.313
**PH**	t-test	-0.071	0.944	1	0.009
**IFS**	t-test	-1.297	0.208	1	0.24
**BA46**	t-test	2.563	**0.017**	0.417	0.525
**BA8**	t-test	0.407	0.688	1	0.065
**IPC**	t-test	1.657	0.111	1	0.278
**Amygdala**	t-test	-0.588	0.562	1	0.094
**Caudate**	t-test	2.619	**0.015**	0.384	0.395

Table S15: Results of prior knowledge dependent, i.e. *(Magic$_{pre}$ > Control$_{pre}$) > (Magic$_{post}$-Control$_{post}$)*, ROI analysis. Data was tested for normality, in case normality was violated, we performed a Wilcoxon signed-rank test, otherwise we performed an undirected t-test for dependent measurements. P-values were Bonferroni-Holm corrected.

ROI	test	statistic	p-val	p-corrected	effect-size
**V1**	t-test	0.039	0.969	1	0.01
**V2**	t-test	1.11	0.278	1	0.302
**V3**	t-test	0.448	0.658	1	0.116
**hV4**	t-test	-1.271	0.216	1	0.386
**V3A**	t-test	1.796	0.086	1	0.441
**V3B**	t-test	-2.223	**0.036**	0.836	0.526
**LO**	Wilcoxon	98	0.143	1	-0.411
**VO**	t-test	-1.718	0.099	1	0.475
**IPS**	t-test	-1.699	0.103	1	0.45
**FEF**	t-test	-1.777	0.089	1	0.487
**IFJ**	t-test	-1.993	0.058	1	0.476
**6r**	t-test	-0.274	0.786	1	0.074
**BA6**	Wilcoxon	111	0.277	1	-0.291
**pdACC**	t-test	-0.125	0.901	1	0.034
**adACC**	t-test	2.74	**0.012**	0.303	0.577
**vACC**	Wilcoxon	42	**0.001**	**0.033**	0.868
**8BM**	t-test	-0.639	0.529	1	0.085
**AI**	t-test	-1.45	0.16	1	0.371
**AVI**	t-test	-2.353	**0.028**	0.689	0.436
**PH**	t-test	-1.101	0.282	1	0.322
**IFS**	t-test	-2.013	0.056	1	0.427
**BA46**	t-test	-0.609	0.548	1	0.159
**BA8**	t-test	-0.953	0.351	1	0.264
**IPC**	t-test	-0.636	0.531	1	0.152
**Amygdala**	t-test	2.252	**0.034**	0.821	0.609
**Caudate**	t-test	-0.345	0.734	1	0.08

Table S16: rmANOVA main effect results for the VOE type condition. Degrees of freedom (df) shown here are the Greenhouse Geisser corrected dfs. Uncorrected dfs were 2 and 46, respectively. P-values were Bonferroni-Holm corrected.

ROI	F-value	df1	df2	p-val	p-corrected	effect-size
**V1**	10.382	1.447	33.28	**0.001**	**0.018**	0.093
**V2**	16.842	1.619	37.23	**< 0.001**	**<0.001**	0.152
**V3**	11.221	1.902	43.757	**< 0.001**	**0.003**	0.117
**hV4**	13.911	1.919	44.143	**< 0.001**	**0.001**	0.149
**V3A**	8.843	1.643	37.798	**0.001**	**0.022**	0.117
**V3B**	8.82	1.687	38.797	**0.001**	**0.021**	0.103
**LO**	24.806	1.998	45.951	**< 0.001**	**<0.001**	0.256
**VO**	18.095	1.675	38.516	**< 0.001**	**<0.001**	0.207
**IPS**	11.649	1.879	43.219	**< 0.001**	**0.002**	0.118
**FEF**	2.391	1.948	44.809	0.104	1	0.025
**IFJ**	11.609	1.951	44.881	**< 0.001**	**0.002**	0.119
**6r**	15.593	1.803	41.471	**< 0.001**	0	0.157
**BA6**	3.635	1.963	45.15	**0.035**	0.387	0.031
**pdACC**	2.269	1.747	40.185	0.123	1	0.02
**adACC**	2.095	1.829	42.06	0.139	1	0.023
**vACC**	0.757	1.652	37.997	0.452	1	0.007
**8BM**	1.774	1.93	44.399	0.183	1	0.019
**AI**	8.526	1.71	39.338	**0.001**	**0.022**	0.091
**AVI**	5.451	1.941	44.648	**0.008**	0.106	0.042
**PH**	32.137	1.811	41.657	**< 0.001**	**<0.001**	0.296
**IFS**	1.939	1.509	34.698	0.167	1	0.025
**BA46**	0.347	1.867	42.945	0.694	1	0.004
**BA8**	1.435	1.737	39.959	0.249	1	0.018
**IPC**	4.538	1.896	43.614	**0.018**	0.212	0.049
**Amygdala**	8.708	1.646	37.847	**0.001**	**0.022**	0.113
**Caudate**	0.599	1.71	39.339	0.529	1	0.007

Table S17: rmANOVA results for interaction between VOE type and revelation condition. Degrees of freedom (df) shown here are the Greenhouse Geisser corrected dfs. Uncorrected dfs were 2 and 46, respectively. P-values were Bonferroni-Holm corrected.

ROI	F-value	df1	df2	p-val	p-corrected	effect-size
**V1**	0.409	1.979	45.517	0.665	1	0.005
**V2**	0.776	1.653	38.021	0.445	1	0.009
**V3**	0.38	1.776	40.841	0.662	1	0.005
**hV4**	0.487	1.781	40.964	0.596	1	0.005
**V3A**	0.445	1.926	44.296	0.636	1	0.005
**V3B**	0.337	1.895	43.594	0.704	1	0.003
**LO**	0.221	1.981	45.566	0.8	1	0.002
**VO**	0.195	1.898	43.665	0.813	1	0.002
**IPS**	2.282	1.987	45.694	0.114	1	0.018
**FEF**	4.033	1.846	42.452	**0.028**	0.748	0.021
**IFJ**	0.753	1.541	35.444	0.446	1	0.006
**6r**	0.125	1.579	36.309	0.835	1	0.001
**BA6**	2.936	1.741	40.032	0.071	1	0.021
**pdACC**	0.66	1.961	45.098	0.519	1	0.011
**adACC**	4.161	1.624	37.354	**0.03**	0.793	0.049
**vACC**	0.946	1.9	43.707	0.392	1	0.014
**8BM**	2.52	1.965	45.203	0.093	1	0.016
**AI**	0.781	1.629	37.468	0.441	1	0.009
**AVI**	1.359	1.72	39.569	0.266	1	0.012
**PH**	0.885	1.697	39.035	0.405	1	0.01
**IFS**	0.32	1.881	43.26	0.715	1	0.003
**BA46**	0.337	1.972	45.362	0.713	1	0.004
**BA8**	0.132	1.922	44.206	0.869	1	0.002
**IPC**	1.295	1.758	40.438	0.282	1	0.012
**Amygdala**	1.797	1.68	38.636	0.184	1	0.028
**Caudate**	1.879	1.813	41.695	0.169	1	0.023

Decoding accuracies and knowledge-dependent differences

Multivariate decoding analyses were performed in a cross-validated fashion, training a linear discriminative analysis (LDA) decoder on data from VOEs performed with two objects (e.g., balls and playing cards) and tested its accuracy on data from VOEs performed with the third object (e.g., pencil). We did this decoding analysis with data before and after revelation of the magic tricks. We report all decoding accuracies and differences in decoding accuracies between pre and post revelation data for each ROI in Tables S18-21.

Table S18: Decoding accuracies of all ROIs pre revelation. An LDA classifier was trained to decode the perceived VOE pre revelation from beta estimates in a ROI. The last two rows show the lower and upper bounds of a 95% confidence interval. Chance level is at 33%.

ROIs	Mean accuracies	95% CI lower	95% CI higher
**V1**	0.441	0.417	0.465
**V2**	0.448	0.424	0.471
**V3**	0.453	0.423	0.483
**hV4**	0.429	0.397	0.461
**V3A**	0.396	0.375	0.417
**V3B**	0.411	0.386	0.435
**LO**	0.428	0.399	0.456
**VO**	0.404	0.371	0.437
**IPS**	0.419	0.392	0.446
**FEF**	0.345	0.318	0.372
**IFJ**	0.367	0.338	0.397
**6r**	0.352	0.327	0.378
**BA6**	0.362	0.336	0.389
**pdACC**	0.354	0.335	0.373
**adACC**	0.35	0.324	0.375
**vACC**	0.362	0.326	0.399
**8BM**	0.338	0.312	0.364
**AI**	0.351	0.336	0.367
**AVI**	0.337	0.314	0.36
**PH**	0.44	0.411	0.469
**IFS**	0.356	0.328	0.385
**BA46**	0.363	0.34	0.387
**BA8**	0.365	0.336	0.394
**IPC**	0.349	0.322	0.376
**Amygdala**	0.317	0.29	0.344
**Caudate**	0.338	0.306	0.37
**3rd-ventricle**	0.352	0.324	0.381

Table S19: Decoding accuracies of all ROIs post revelation. A LDA classifier was trained to decode the perceived VOE pre revelation from beta estimates in a ROI. The last two rows show the lower and upper bounds of a 95% confidence interval. Chance level is at 33%.

ROIs	Mean accuracies	95% CI lower	95% CI higher
**V1**	0.38	0.347	0.413
**V2**	0.386	0.359	0.413
**V3**	0.399	0.372	0.426
**hV4**	0.394	0.366	0.422
**V3A**	0.381	0.36	0.401
**V3B**	0.367	0.339	0.395
**LO**	0.36	0.337	0.383
**VO**	0.384	0.36	0.407
**IPS**	0.357	0.332	0.382
**FEF**	0.341	0.321	0.36
**IFJ**	0.338	0.311	0.365
**6r**	0.329	0.302	0.356
**BA6**	0.355	0.327	0.383
**pdACC**	0.35	0.324	0.375
**adACC**	0.347	0.325	0.368
**vACC**	0.354	0.328	0.379
**8BM**	0.341	0.319	0.362
**AI**	0.347	0.325	0.369
**AVI**	0.349	0.324	0.374
**PH**	0.369	0.341	0.396
**IFS**	0.319	0.295	0.344
**BA46**	0.337	0.313	0.361
**BA8**	0.31	0.284	0.336
**IPC**	0.337	0.315	0.36
**Amygdala**	0.324	0.299	0.349
**Caudate**	0.336	0.316	0.357
**3rd-ventricle**	0.318	0.292	0.343

Table S20: Paired t-tests comparing decoding accuracies when training and testing in pre vs post revelation data for ROIs with significant decoding accuracies before revelation (corrected for multiple comparisons using a Bonferroni-Holm correction).

ROIs	T	p-unc	p-corr	Cohen's-d
**V1**	3.173	**0.002**	**0.011**	0.849
**V2**	4.611	**< 0.001**	**0.001**	0.99
**V3**	3.805	**< 0.001**	**0.004**	0.756
**hV4**	1.717	**0.05**	0.149	0.464
**V3A**	0.934	0.18	0.265	0.289
**V3B**	2.297	**0.016**	0.062	0.674
**LO**	3.661	**0.001**	**0.005**	1.051
**VO**	1.143	0.132	0.265	0.284
**IPS**	3.845	**< 0.001**	**0.004**	0.956
**PH**	3.568	**0.001**	**0.005**	1.009

Table S21: Paired t-tests comparing decoding accuracies when training and testing over objects in pre vs. post revelation data for ROIs with non-significant decoding before revelation (corrected for multiple comparisons using a Bonferroni-Holm correction).

ROIs	T	p-unc	p-corr	Cohen's-d
**FEF**	0.249	0.403	1	0.068
**IFJ**	1.382	0.09	1	0.417
**6r**	1.224	0.117	1	0.354
**BA6**	0.402	0.346	1	0.111
**pdACC**	0.275	0.393	1	0.083
**adACC**	0.185	0.427	1	0.049
**vACC**	0.413	0.342	1	0.11
**8BM**	-0.182	0.571	1	0.049
**AI**	0.293	0.386	1	0.097
**AVI**	-1.104	0.86	1	0.204
**IFS**	2.078	**0.025**	0.393	0.556
**BA46**	1.505	0.073	1	0.439
**BA8**	3.304	**0.002**	**0.026**	0.79
**IPC**	0.684	0.25	1	0.188
**Amygdala**	-0.355	0.637	1	0.108
**Caudate**	0.084	0.467	1	0.026
**3rd-ventricle**	1.675	0.054	0.806	0.508

MVPA control analyses

In our main MVPA analysis, we could show that decoding accuracies of magic tricks significantly dropped in posterior sensory areas after revealing the method behind the magic tricks shown. To rule out that any differences in decoding accuracies before and after explanation of the tricks are due to time confounds (e.g., decreased alertness), we performed a control analysis using data from the matched control videos showing similar actions, but no VOE. Using estimates from the control videos, we performed three two-label classifications (appear/change, change/vanish and vanish/appear), separately for data before and after the explanation of the tricks. Importantly, visual input during the vanish and change control videos always include a visible red object, while visual content during the appear control videos generally include no object (except one video performed with a ball). Accordingly, if linear classifiers would capture information based on visual differences between the discrete moments of the videos, we expect to see above-chance decoding when classifying estimates of control videos between the appear and change conditions and between the appear and vanish conditions, as classification is based on videos either showing a red object (change and vanish control videos) and videos showing no object (appear control videos). In contrast, we expect chance-level decoding accuracies when classifying estimates based on control videos from the change and vanish conditions, as a red object is present in both control videos. Moreover, we hypothesized that if time confounds affected decoding (e.g., decreased alertness), this would also be evident in this control analysis, reflected in differences between decoding accuracies from data before and after explanation. Since we only had half the amount of video presentations in the control compared to the magic condition, we run an extra decoding analysis using half of the magic data (using estimates from video presentations that were not flipped in the first run and flipped in the second run, to ensure using visually different videos for decoding) in a comparable two-label classification (appear/vanish). In contrast to the control MVPA analyses based on the matched control videos, we did not perform a two-label classification based on data from appear and change magic videos, as they involve stimuli that show either a red object (appear magic videos) or a blue object (change magic videos) (see Figure S9 and Table S22-23).

None of the ROIs showed significant decoding in the control classification using estimates from color change and vanish *control videos* (see Figure S9D). This was expected as the content of both control videos were similar (i.e., the magician showing a red object). Two-way classification using data from appear and color change control videos revealed significant above chance (50%) decoding in most ROIs before and after revelation (except for V3B and VO pre-revelation and hV4, V3B, LO, VO, IPS and PH post-revelation – see Figure S9C and Table S23). Finally, in the two-label control classification of appear and vanish control videos, significant decoding was possible in visual areas V1, V2, V3, hV4 and LO using pre-revelation data and in hV4, V3A, LO and PH using post-revelation data (see Figure S9B and Table S23). A significant difference in decoding accuracies between pre and post revelation was found only in V1. While these results show that different visual content is decodable in posterior visual areas, they contrast to the two-label classification of appear and vanish *magic videos*, in which we systematically observed significant above-chance decoding in all tested areas before the explanation of the magic tricks, but not after the explanation of the tricks. Significant differences between before and after revelation decoding accuracies were found in V1, V2, V3 and V3A (see Figure S9A and Table S23). Together, these results suggest that the differences we observe when decoding VOE before and after explanation of the tricks

cannot be attributed to a time confound, as these differences were not observable within the matched control videos.

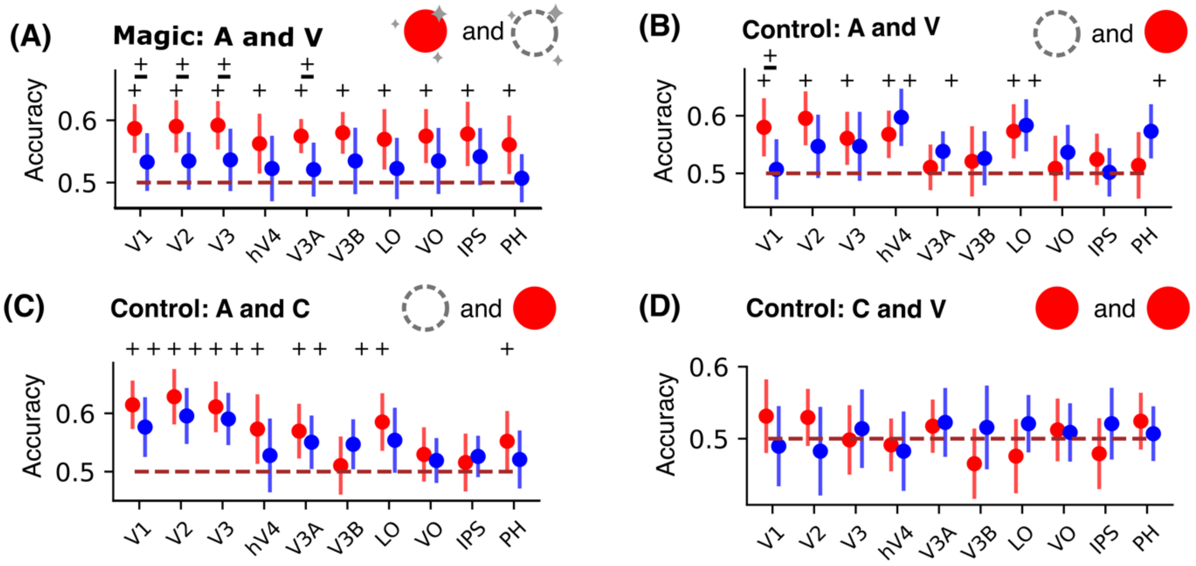

Figure S9. Decoding accuracies and differences between accuracies using data before vs. after revelation. **(A)** using data of magic videos showing objects appearing (i.e., a red object is in the scene) and objects vanishing (i.e., no object is in the scene); **(B)** using data of control videos for appear magic tricks (i.e., no object is in the scene) and control videos for vanish magic tricks (i.e., a red object is in the scene); **(C)** using data of control videos for appear magic tricks (i.e., no object is in the scene) and control videos for color change magic tricks (i.e., a red object is in the scene), and **(D)** using data of control videos for color change magic tricks and control videos for vanish magic tricks (i.e., a red object is both scenes). Results show significant above chance decoding in all visual ROIs using data from magic tricks before revelation, but not after revelation. Importantly, we observe significant differences in decoding accuracies before vs. after revelation in early visual areas V1-V3 and V3A. Decoding accuracies from control videos do not change as a factor of prior knowledge (except for V1 in B).

Table S22: Comparing two label decoding accuracies – appear vs vanish – using half the data of our magic condition pre and post revelation in ROIs with significant decoding accuracies before revelation (corrected for multiple comparisons using a Bonferroni-Holm correction).

ROIs	T	p-unc	p-corr	Cohen's-d
**V1**	2.099	**0.023**	0.235	0.529
**V2**	1.799	**0.043**	0.341	0.53
**V3**	1.721	**0.049**	0.345	0.523
**hV4**	1.172	0.127	0.403	0.334
**V3A**	2.099	**0.023**	0.235	0.626
**V3B**	1.604	0.061	0.367	0.428
**LO**	1.196	0.122	0.403	0.405
**VO**	1.315	0.101	0.403	0.348
**IPS**	1.017	0.16	0.403	0.315
**PH**	1.593	0.062	0.367	0.528

Table S23: Comparing two label decoding accuracies using data from the matched control condition pre and post revelation in ROIs with significant decoding accuracies before revelation (corrected for multiple comparisons using a Bonferroni-Holm correction). None of the ROIs showed differences between pre and post revelation decoding, with exception of V1 in the Appear vs. Vanish (shown in bold).

ROIs	T	p-unc	p-corr	Cohen's-d
**Control videos: Appear – Vanish**				
**V1**	2.209	**0.019**	0.187	0.599
**V2**	1.51	0.072	0.65	0.402
**V3**	0.399	0.347	1	0.11
**hV4**	-0.959	0.826	1	0.273
**V3A**	-1.088	0.856	1	0.315
**V3B**	-0.165	0.565	1	0.04
**LO**	-0.358	0.638	1	0.095
**VO**	-0.756	0.771	1	0.224
**IPS**	0.758	0.228	1	0.22
**PH**	-1.942	0.968	1	0.473
**Control videos: Change – Appear**				
**V1**	1.346	0.096	0.765	0.345
**V2**	1.499	0.074	0.688	0.292
**V3**	0.835	0.206	1	0.199
**hV4**	1.538	0.069	0.688	0.31
**V3A**	0.653	0.26	1	0.174
**V3B**	-1.032	0.844	1	0.331
**LO**	1.166	0.128	0.895	0.251
**VO**	0.379	0.354	1	0.103
**IPS**	-0.413	0.658	1	0.102
**PH**	1.013	0.161	0.965	0.259
**Control videos: Vanish – Change**				
**V1**	1.138	0.133	1	0.329
**V2**	1.514	0.072	0.718	0.382
**V3**	-0.512	0.693	1	0.128
**hV4**	0.247	0.404	1	0.078
**V3A**	-0.18	0.571	1	0.051
**V3B**	-1.363	0.907	1	0.396
**LO**	-1.313	0.899	1	0.413
**VO**	0.115	0.455	1	0.035
**IPS**	-1.486	0.925	1	0.355
**PH**	0.622	0.27	1	0.189

References

Brass, M., Schmitt, R. M., Spengler, S., & Gergely, G. (2007). Investigating Action Understanding: Inferential Processes versus Action Simulation. *Current Biology*, *17*(24), 2117–2121. https://doi.org/10.1016/j.cub.2007.11.057

Danek, A. H., Öllinger, M., Fraps, T., Grothe, B., & Flanagin, V. L. (2015). An fMRI investigation of expectation violation in magic tricks. *Frontiers in Psychology*, *6*(FEB), 1–11. https://doi.org/10.3389/fpsyg.2015.00084

Deen, B., Koldewyn, K., Kanwisher, N., & Saxe, R. (2015). Functional Organization of Social Perception and Cognition in the Superior Temporal Sulcus. *Cerebral Cortex*, *25*(11), 4596–4609. https://doi.org/10.1093/cercor/bhv111

Fischl, B., Salat, D. H., Busa, E., Albert, M., Dieterich, M., Haselgrove, C., van der Kouwe, A., Killiany, R., Kennedy, D., Klaveness, S., Montillo, A., Makris, N., Rosen, B., & Dale, A. M. (2002). Whole Brain Segmentation. *Neuron*, *33*(3), 341–355. https://doi.org/10.1016/S0896-6273(02)00569-X

Geuter, S., Qi, G., Welsh, R. C., Wager, T. D., & Lindquist, M. A. (2018). *Effect Size and Power in fMRI Group Analysis*. https://doi.org/10.1101/295048

Glasser, M. F., Coalson, T. S., Robinson, E. C., Hacker, C. D., Harwell, J., Yacoub, E., Ugurbil, K., Andersson, J., Beckmann, C. F., Jenkinson, M., Smith, S. M., & Van Essen, D. C. (2016). A multi-modal parcellation of human cerebral cortex. *Nature*, *536*(7615), 171–178. https://doi.org/10.1038/nature18933

Grill-Spector, K., Henson, R., & Martin, A. (2006). Repetition and the brain: Neural models of stimulus-specific effects. *Trends in Cognitive Sciences*, *10*(1), 14–23. https://doi.org/10.1016/j.tics.2005.11.006

Hiser, J., & Koenigs, M. (2018). The Multifaceted Role of the Ventromedial Prefrontal Cortex in Emotion, Decision Making, Social Cognition, and Psychopathology. *Biological Psychiatry*, *83*(8), 638–647. https://doi.org/10.1016/j.biopsych.2017.10.030

Jastorff, J., Clavagnier, S., Gergely, G., & Orban, G. A. (2011). Neural Mechanisms of Understanding Rational Actions: Middle Temporal Gyrus Activation by Contextual Violation. *Cerebral Cortex*, *21*(2), 318–329. https://doi.org/10.1093/cercor/bhq098

Krekelberg, B., Boynton, G. M., & Van Wezel, R. J. A. (2006). Adaptation: From single cells to BOLD signals. *Trends in Neurosciences*, *29*(5), 250–256. https://doi.org/10.1016/j.tins.2006.02.008

Marsh, L. E., Mullett, T. L., Ropar, D., & Hamilton, A. F. D. C. (2014). Responses to irrational actions in action observation and mentalising networks of the human brain. *NeuroImage*, *103*, 81–90. https://doi.org/10.1016/j.neuroimage.2014.09.020

Parris, B. A., Kuhn, G., Mizon, G. A., Benattayallah, A., & Hodgson, T. L. (2009). Imaging the impossible: An fMRI study of impossible causal relationships in magic tricks. *NeuroImage*, *45*(3), 1033–1039. https://doi.org/10.1016/j.neuroimage.2008.12.036

Saxe, R., & Kanwisher, N. (2003). People thinking about thinking peopleThe role of the temporo-parietal junction in "theory of mind." *NeuroImage*, *19*(4), 1835–1842. https://doi.org/10.1016/S1053-8119(03)00230-1

Szucs, D., & Ioannidis, J. Pa. (2020). Sample size evolution in neuroimaging research: An evaluation of highly-cited studies (1990–2012) and of latest practices (2017–2018) in high-impact journals. *NeuroImage*, *221*, 117164. https://doi.org/10.1016/j.neuroimage.2020.117164
